# Borophosphate glass as an active media for CuO nanoparticle growth: an efficient catalyst for selenylation of oxadiazoles and application in redox reactions

**DOI:** 10.1038/s41598-020-72129-w

**Published:** 2020-09-17

**Authors:** Marcos R. Scheide, Marcos M. Peterle, Sumbal Saba, José S. S. Neto, Guilherme F. Lenz, Rosane Dias Cezar, Jorlandio F. Felix, Giancarlo V. Botteselle, Ricardo Schneider, Jamal Rafique, Antonio L. Braga

**Affiliations:** 1 Departamento de Química, Universidade Federal de Santa Cararina - UFSC, Florianópolis, SC 88040-900 Brazil; 2 Centro de Ciências Naturais e Humanas-CCNH, Universidade Federal do ABC, Santo André, SP 09210-580 Brazil; 3 Departamento de Engenharias e Exatas, Universidade Federal do Paraná - UFPR, Palotina, PR 85950-000 Brazil; 4 Instituto de Química, Universidade Federal do Mato Grosso do Sul - UFMS, Campo Grande, MS 79074-460 Brazil; 5 Instituto de Física, Núcleo de Física Aplicada, Universidade de Brasília - UNB, Brasília, DF 70910-900 Brazil; 6 Centro de Engenharias e Ciências Exatas (CECE), Universidade Estadual do Oeste do Paraná - UNIOESTE, Toledo, PR 85903-000 Brazil; 7 Group of Polymers and Nanostructures, Universidade Tecnológica Federal do Paraná - UTFPR, Toledo, PR 85902-490 Brazil

**Keywords:** Nanoparticle synthesis, Catalysis, Catalyst synthesis, Heterogeneous catalysis, Organic chemistry, Synthetic chemistry methodology, Green chemistry

## Abstract

Herein, we report the preparation of CuO@ borophosphate nanoparticles (CuOnano@glass) and their wide catalytic applications. The glass annealing, under a controlled atmosphere, enables the growth of copper nanoparticles on the glass surface (not within) by an uncommon bottom-up process. Following the thermal annealing of metallic nanoparticles under air atmosphere, supported copper oxide nanoparticles CuONPs on the glass surface can be obtained. The approach enables the glass matrix to be explored as a precursor and a route for the synthesis of supported copper-based nanoparticles in a solvent-free process without immobilization steps or stabilizing agents. In order to demonstrate the wide synthetic utility of this CuONPs glass-based catalyst, one-pot three-component domino reactions were performed under an air atmosphere, affording the desired selenylated oxadiazoles in good to excellent yields. We also extended the application of these new materials as a glass-based catalyst in the phenol hydroxylation and the reduction of 4-nitrophenol.

## Introduction

Glass materials are widely used in everyday life, with a range of applications from the technological (e.g., lasers) to the ordinary, such as kitchen devices, bottles and windows in buildings. Notable characteristics are the low reactiveness and high stability of the oxide glasses, mainly silica-based glasses, upon exposure to a variety of chemicals. Due to the well-established route for the synthesis of glass, glass-based materials have gained great importance in our lives^1^.

Phosphate-based (borophosphate) glasses show remarkable characteristics including low processing temperatures, simple starting materials and high capacity to dissolve metal oxides^2^. In addition, they can be easily doped with copper or silver ions^2,3^. In particular, copper-doped borophosphate glass enables the synthesis of supported nanoparticles through annealing under a hydrogen atmosphere by a bottom-up process with high Surface-Enhanced Raman Scattering (SERS) activity^4^. The growth of metallic nanoparticles on the glass surface (not within), with short annealing times, is unusual^5^ and the growth of supported nanoparticles on annealing under a reductive atmosphere can be exploited for the development of a glass-based catalyst. The synthesis and immobilization in a single-step process, without organic/inorganic reducing agents (e.g., citric acid, ascorbate, formaldehyde, hydrazine and hydrides) produces nanoparticles without the presence of stabilizers and/or ligands on the glass surface.

The development of new approaches or new types of catalysts using low-cost and abundant metals that can be obtained by classical methods and in large scale is highly desirable. In this regard, glass has good potential, but the use of glass materials as catalysts is relatively rare. Glass materials are recognized for their chemical resistance and non-reactive character, notably the silica-based glasses, and their use to produce laboratory glassware is widespread. As a consequence, glass materials are frequently applied as a support material. Thus, the copper-doped borophosphate glass with metallic copper nanoparticles grown on the glass surface can be oxidized to obtain copper-based nanoparticles, i.e., copper oxide nanoparticles (CuONPs), for catalytic applications.

The application of nanoparticles as a catalyst in organic transformations has gained considerable attention because such catalysts are associated with a more effective process and compete favourably with classical methodologies^6–11^. In this regard, CuONPs have been used extensively in the formation of new C–C and C–heteroatom bonds as well as in C($\hbox {sp}^2$)–H bond functionalization^12,13^. The formation of C–Se bonds is gaining increasing interest due to the subsequent biological properties^14–21^. In relation to current pandemic of COVID19, a very interesting study just appeared in the literature where ebselen (organoselenium compound) showed a pronounced antiviral effect in the treatment of Vero Cells infected with COVID19^22^. Similarly, the 1,3,4-oxadiazole (ODZ) scaffold is an interesting heterocycle. It is used in pharmaceuticals and considered a “privileged structure”^23–26^. Considering the biological relevance of organoselenides and the therapeutic properties of ODZs, few methods are available to access selenylated ODZs^27–30^.

Phenolic and nitro compounds are common organic pollutants found in industrial waste water. Specifically, phenol and 4-nitrophenol are among the effluents that are encountered abundantly due to their widespread industrial uses. While the emission in the environment of 4-nitrophenol is usually from the pesticide and pharmaceutical industry, the main source of phenol is the petrochemical activity. Furthermore, phenol and 4-nitrophenol are used as raw chemicals/intermediaries in the original processes for the production of paracetamol^31^.

To the best of our knowledge, the application of CuONPs as a catalyst in the cross-coupling reaction for the chalcogenation of ODZs has not yet been explored. Thus, in connection with our continuing interest in designing new materials^32–34^ and developing eco-friendly processes for cross-coupling reactions^35–40^, herein we report the synthesis of CuO supported on borophosphate glass and its application as an efficient catalyst in the synthesis of selenylated 1,3,4-oxadiazoles. The method involves the use of elemental Se and iodoarenes, in DMSO, under an open-to-air atmosphere. Furthermore, the performance of CuOnano@glass catalyst was also evaluated in the phenol oxidation by $\hbox {H}_{2}\hbox {O}_{2}$ and the reduction of 4-nitrophenol by $\hbox {NaBH}_{4}$.

## Materials and methods

### Glass synthesis and nanoparticle growth

The borophosphate glass samples were synthesized using a $\hbox {NaH}_{2}\hbox {PO}_{4}$–$\hbox {H}_{3}\hbox {BO}_{3}$–$\hbox {Al}_{2}\hbox {O}_{3}$ glass matrix with a $\hbox {NaH}_{2}\hbox {PO}_{4}$ to $\hbox {H}_{3}\hbox {BO}_{3}$ ratio of 2 (by mol%), while $\hbox {Al}_{2}\hbox {O}_{3}$ was added to the matrix in proportions of (mol%) 10 and 3 $\hbox {Cu}_{2}\hbox {O}$ (6 mol% copper ions). In a typical synthesis route, 5 g of the above-mentioned raw chemical compounds were mixed using an agate mortar for 10 min. The powder mixture was then transferred to a covered Pt/Au crucible (30 mL) and fused for 1 h at 1,050 $^{\circ }\hbox {C}$ in a preheated resistive oven. The glass samples were obtained by quenching them from the melt temperature to room temperature in a graphite mold. The bulk glass samples were crushed and sieved (325–400 mesh range). The powdered copper-doped glass samples were used for the nanoparticle growth and catalysis. The metallic copper nanoparticles (CuNPs) were obtained on the copper-doped glass surface by thermal treatment at $430\ ^{\circ }\hbox {C}$, with a constant flow (150 mL/min) of hydrogen gas (5.0 grade) for 15 min, followed by cooling at room temperature ($\approx \ 25\ ^{\circ }\hbox {C}$) under a hydrogen atmosphere. The CuNPs were then oxidized to give copper oxide nanoparticles (CuONPs) by thermal annealing in a preheated oven at $400\ ^{\circ }\hbox {C}$ for 60 min under air atmosphere.

### Sample characterization

A Bruker D8 Discover diffractometer equipped with Cu $\hbox {K}_{\alpha }$ radiation ($\lambda = 1.5418$ Å) was used, with angles between $30^{\circ }$ and $80^{\circ }$ ($\theta --2\theta$), for the crystallographic characterization of the copper-based nanostructures obtained from the annealing under hydrogen or air atmosphere. The Raman analysis was performed using a Horiba micro-Raman system, model LabRAM HR Evo, with laser power 10 mW, 405 nm excitation wavelength and CCD detector, with additional sample preparation. Deconvolution of the Raman spectrum was carried out using Voigt functions of the Fityk program (version 1.3.1). A PG Instruments spectrometer (model T80+) with a step of 0.5 nm and air as the baseline was used to obtain solid state ultraviolet visible (UV–Vis) spectra. Glass slices doped with 6 mol% of copper ions, with a thickness of 0.1 mm, were scanned before and after the annealing at $430\ ^{\circ }\hbox {C}$ under $\hbox {H}_{2}$ (g) atmosphere. The scanning electron microscopy (SEM) was performed with a JEOL JSM-6390LV scanning electron microscope. In this analysis, CuOnano@glass powder was spread on gold-coated double-sided carbon tape and analyzed using an acceleration voltage of 10 kV. The total content of copper on borophosphate glasses was determined by Inductively Coupled Plasma Optical Emission Spectrometry (ICP-OES) using a Thermo Scientific iCap 6000 Series Spectrometer^2^ using the Cu emission line at 324.754 nm (axial view).

### Synthesis of the seleno 1,3,4-oxadiazole

The appropriate oxadiazole **1** (0.5 mmol), the respective iodo-(hetero)arene **2** (1.0 mmol), selenium powder—325 mesh (79.0 mg, 1.0 mmol), $\hbox {K}_{2}\hbox {CO}_{3}$ (138.2 mg, 1.0 mmol) and CuOnano@glass (15.0 mg, 2.83 mol% of Cu) were placed in a Schlenk tube containing DMSO (2 mL). The reaction was heated to $120\ ^{\circ }\hbox {C}$ in an oil bath for 12 h, with continuous stirring. The mixture was then diluted with ethyl acetate (15 mL) and washed with a saturated solution of NaCl ($3 \times 10\ \hbox {mL}$). The organic phase was separated, dried over $\hbox {MgSO}_{4}$, and concentrated under vacuum. The residue was purified by column chromatography and eluted with a mixture of hexane/ethyl acetate. The identity and purity of the products were confirmed by melting point, $^1\hbox {H}$ NMR, $^{13}\hbox {C}$ NMR, IR and HRMS (see Supplementary Electronic Information).

### Phenol hydroxilation

Hydroxylation of phenol was carried out using CuOnano@glass as catalyst and hydrogen peroxide ($\hbox {H}_{2}\hbox {O}_{2}$) 30 wt% as oxidant^41^. For all tests, 1 mmol of phenol ($\approx \ 94\ \hbox {mg}$) was added to a glass round bottom flask, dissolved in 17 mL of distilled water and kept under agitation at $50\ ^{\circ }\hbox {C}$ during 2 h. The effect of phenol:$\hbox {H}_{2}\hbox {O}_{2}$ molar ratio was investigated in proportions of 1:1, 1:4, 1:7 and 1:10, using 75 mg of CuOnano@glass as catalyst. The oxidant was added in 4 equal portions in the beginning and with 15, 30 and 45 mins of the reaction. The effect of CuOnano@glass mass was investigated using 25, 50, 75, and 100 mg. Phenol conversion was analysed by High-performance liquid chromatography (HPLC) using a Thermo Scientific Ultimate 3000 equipment. The separation was performed at $30\ ^{\circ }\hbox {C}$ using an octadecylsilane C18 column (Ace ltd.), flow rate $0.8\ \hbox {mL min}^{-1}$, with mobile phase composed by the mixture of water and methanol (Panreac HPLC grade) 1:1. The detection performed by a diode-array detector (DAD) at 280 nm. Aliquots of the reaction media were collected at the beginning, during and at the end of the reaction, diluted 10 times with mobile phase and filtered in a 0.22 mm hydrophilic PVDF syringe filter. Analytical standards (Sigma Aldrich, Supelco) were used as reference for sample concentration determination.

### Catalytic measurements of 4-nitrophenol reduction

A predetermined mass of CuOnano@glass catalyst was added to a standard quartz cuvette, a pathlength of 1 cm, with 1 mL of distilled water. A solution of $\hbox {NaBH}_{4}$ (1 mL, 3 mM) and 4-nitrophenol (1 mL, 0.12 mM) was homogenized for 5 mins and added to catalyst in the cuvette which was placed in Thermo Scientific spectrometer (Genesys 10UV Scanning). The reaction progress was measured in the 250–500 nm wavelength range.

## Results and discussion

### Catalyst characterization

**Figure 1:**
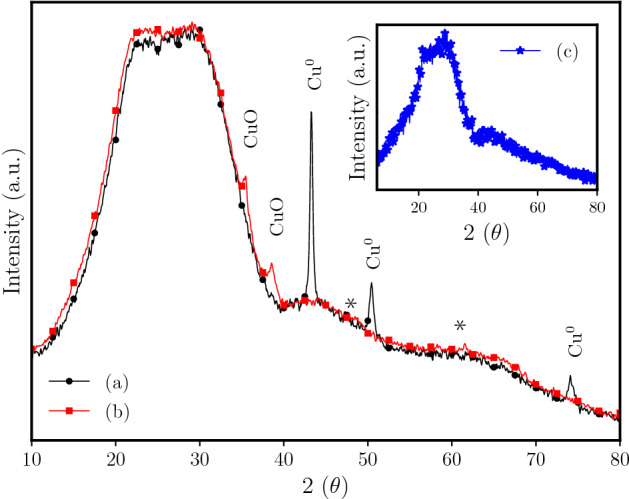
Powder X-ray diffraction analysis of copper-doped borophosphate glass annealed under (a) $\hbox {H}_{2}$ (g) gas at 430 $^{\circ }\hbox {C}$ for 15 min (JCPDS file ($\hbox {Cu}^0$): 04-0836, (b) metallic copper nanoparticles [sample (a)] annealed under air atmosphere for 1 h at $400\ ^{\circ }\hbox {C}$ (JCPDS file (CuO): 80-1917. Graph in insert: (c) borophosphate glass doped without annealing (as-prepared glass sample).

In order to identify the effect of annealing on the phases obtained under hydrogen and air atmosphere, the samples were analyzed by powder X-ray diffraction (PXRD). Figure 1a shows the diffractogram of the copper-doped borophosphate glass after thermal annealing under reductive ($\hbox {H}_{2}$ (g)) atmosphere. The presence of three characteristic XRD peaks at $43.3^\circ$, $50.4^\circ$, and $74.1^\circ$ associated with crystalline planes (111), (200) and (220), respectively, confirm the crystallinity of the metallic copper nanoparticles (JCPDS File 04-0836). The CuO nanoparticle catalyst was obtained on the glass surface through the annealing of the metallic nanoparticles under air atmosphere. In this way, under oxidative conditions the metallic copper can be converted to copper oxide nanoparticles (CuONPs). The CuO phase was confirmed by XRD peaks at 35.3 (111) and 38.6 (111) (JCPDS file:80-1917) (Fig. 1b). The peaks normally observed at around $48^\circ$ (202) and $61^\circ$ (113), marked in Fig. 1b, show low intensity. The Cu-doped unannealed glass sample (inset Fig. 1c) shows only a broad band characteristic of glass-based materials^42^.

**Figure 2:**
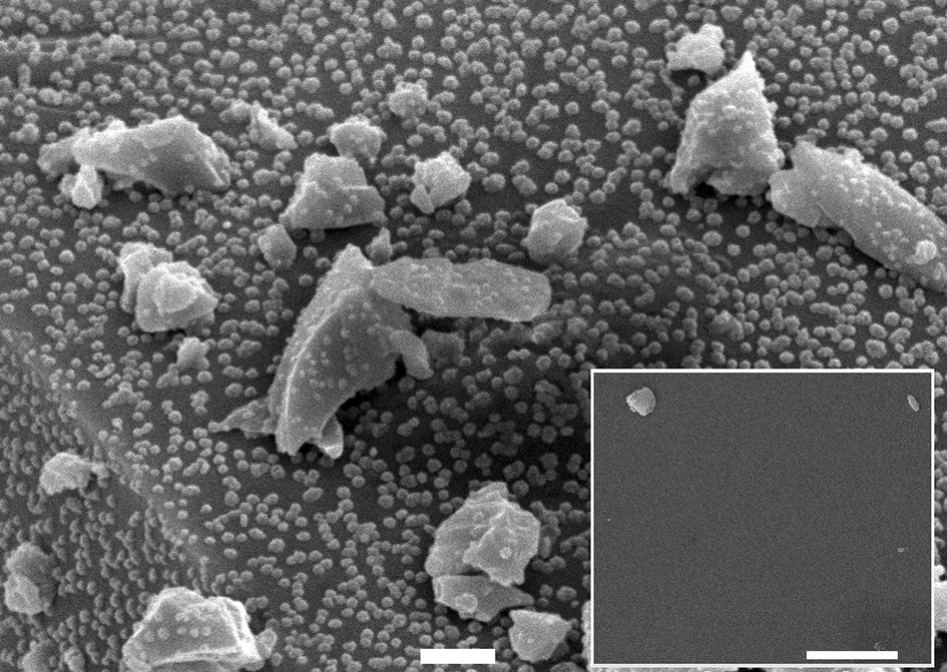
SEM images of CuO nanostructures supported on 6 mol% ($\hbox {Cu}^{2+}$ ions) copper-doped borophosphate glass annealed for 60 min under air atmosphere (main scale bar $1\ \mu \hbox {m}$). Picture in insert: unannealed glass sample (scale bar $1\ \mu \hbox {m}$).

Figure 2 shows the SEM surface analysis of the copper-doped borophosphate glass samples. The nanoparticle growth is thermally activated. Nevertheless, the nanoparticle growth can be accomplished within a short time, e.g., 15 min at $430\ ^{\circ }\hbox {C}$, if it is annealed under a pure hydrogen atmosphere. The metallic nanoparticles can be converted to copper oxide with the annealing under an oxidative atmosphere. Figure 2 shows the SEM images of copper oxide nanoparticles obtained after annealing CuNPs for 60 min under air atmosphere. The copper oxide nanoparticles show spherical-like geometries, homogeneously distributed on the surface of the glass powder.

**Figure 3:**
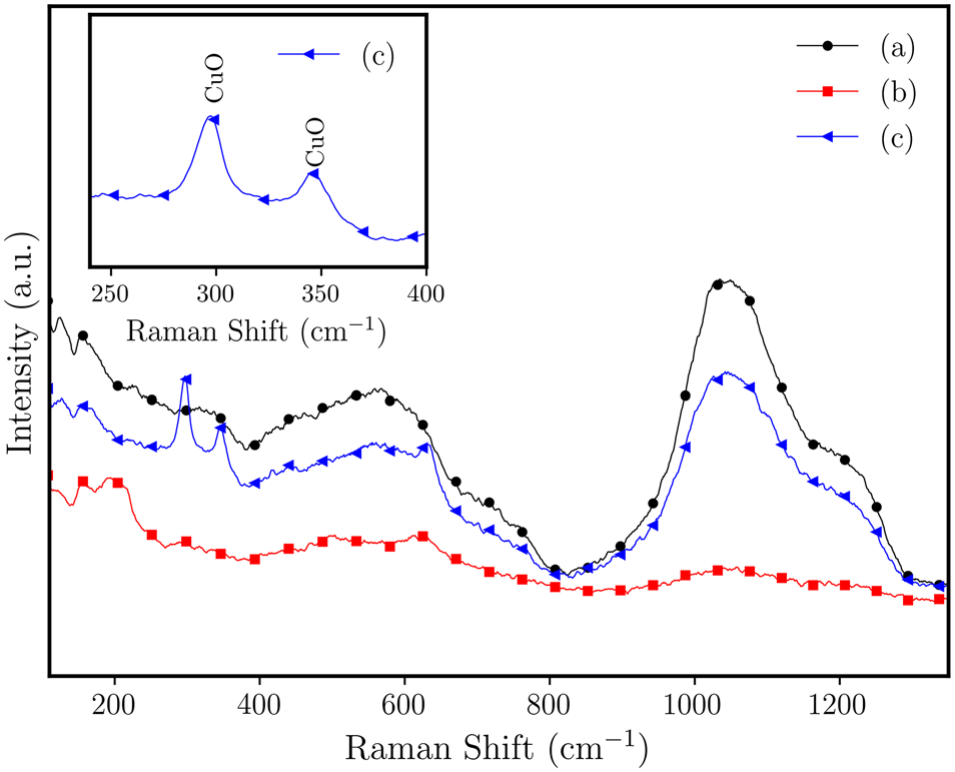
Raman spectra for the copper-doped (6 mol% copper ions) borophosphate glass samples with $\hbox {Al}_{2}\hbox {O}_{3}$ (10 mol%): (a) unannealed (as-prepared), (b) annealed at 430 $^{\circ }\hbox {C}$ under hydrogen for 45 min; and (c) sample (b) with further oxidation, annealing under air atmosphere for 60 min. Graph in insert: detail of Raman spectrum for sample (c) for region of CuO Raman-active modes.

The Raman spectrum of undoped borophosphate glass samples shows two broad bands located below and above $\approx 810\ \hbox {cm}^{-1}$. The most intense band is located between $810\ \hbox {cm}^{-1}$ and $1320\ \hbox {cm}^{-1}$^32,43^. The addition of copper ions does not change considerably the profile of the Raman spectra^3,44^. The broad Raman band between 810 and $1320\ \hbox {cm}^{-1}$ (Fig. 3a) is assigned to Raman scattering of the complex overlap of $\hbox {Q}^0$ ($\hbox {PO}_{4}^{3-}$), $\hbox {Q}^1$(P–O stretching vibrations within the end groups), and $\hbox {Q}^2$ (symmetric/asymmetric stretching of $\hbox {PO}_{2}$) units^45,46^.

Figure 3b shows the Raman spectrum for the annealed copper-doped borophosphate glass samples. The annealing under reducing atmosphere promotes the growth of crystalline metallic copper nanoparticles (Fig. 3a) that are Raman inactive. The oxidized sample shows two peaks ($296\ \hbox {cm}^{-1}$ and $345\ \hbox {cm}^{-1}$ ($\Delta = 49\ \hbox {cm}^{-1}$) (inset of Fig. 3c) at the lower Raman shift. We attribute the peaks to the Raman-active modes of the CuO phase, noted in the PXRD analysis (Fig. 1b). The Raman shift is in agreement with the active modes ($\hbox {A}_{\mathrm{g}}$ ($296\ \hbox {cm}^{-1}$) and $\hbox {B}_{\mathrm{g}}$ ($346\ \hbox {cm}^{-1}$) symmetry ($\Delta = 50\ \hbox {cm}^{-1}$), respectively) of CuO, reported by Debbichi et al.^47^.

**Figure 4:**
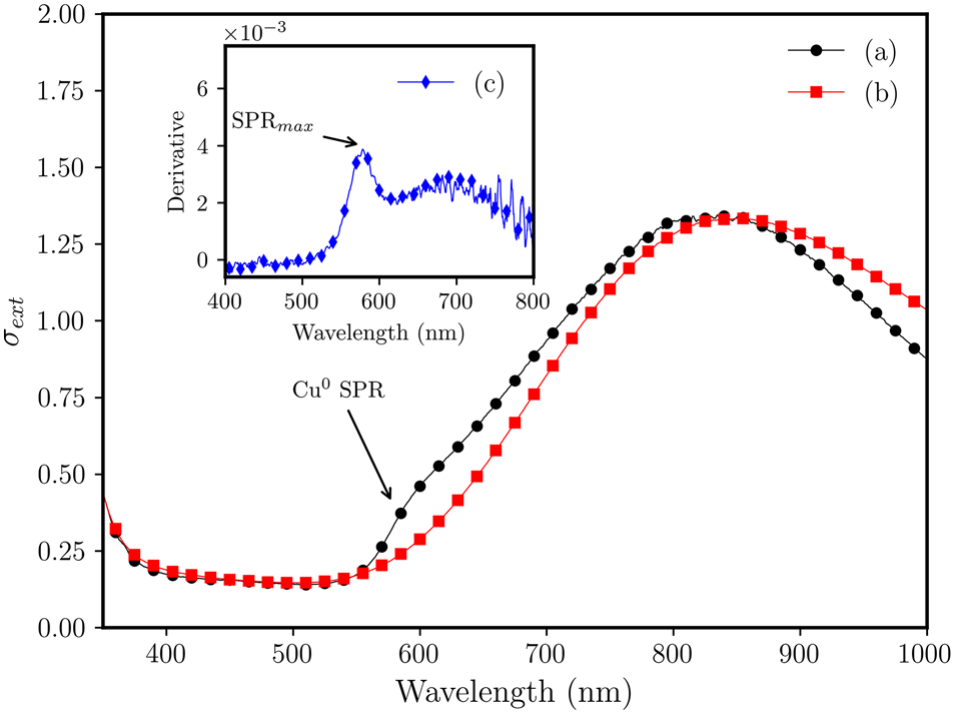
UV–Vis spectra for $\hbox {Al}_{2}\hbox {O}_{3}$ (10 mol%) Cu-doped glass: (a) 45 min at $430\ ^{\circ }\hbox {C}$ under $\hbox {H}_{2}$ (g) atmosphere, (b) unannealed glass sample. Inset: (c) the derivative of the curve of sample (a) showing the maximum of surface plasmon resonance ($\hbox {SPR}_{max}$) peak.

Figure 4 shows the UV–Vis spectra obtained for the copper-doped annealed glass and unannealed glass samples. A characteristic surface plasmon resonance (SPR) band can be observed at $\approx 579\ \hbox {nm}$ (Figure 4c), which is attributed to CuNPs obtained on the glass surface by annealing under hydrogen atmosphere^5^. For the undoped glass (Fig. 4b) the SPR band was not observed.

### Direct C($\hbox {sp}^2$)–H bond selenylation of oxadiazoles

In order to check the applicability of the new material, it was tested as a catalyst in a three-component reaction to access selenylated-oxadiazoles. Firstly, we carried out studies to ascertain the optimal conditions. For this purpose, we selected 0.5 mmol of 2-(4-tolyl)-[1,3,4]-oxadiazole **1a**, elemental selenium (2 molar equiv.) and iodobenzene (2 molar equiv.) **2a** as model substrates. The catalytic potential of the nano CuO supported on borophosphate glass was screened under various reaction conditions (Table S1).

The focus of the preliminary experiments was to optimize the catalyst loading and mesh size. Thus, CuOnano@glass loadings of 10.0 mg to 17.5 mg (entries 1–4) were evaluated and the best results were obtained with 15.0 mg (2.8 mol% of Cu, entry 3). There was no formation of the product when the reaction was conducted in the absence of catalyst (entry 5) or borophosphate glass without CuO (entry 6). Next, we investigated the importance of the mesh size of the catalyst (entry 7–8). The reaction lost efficiency when the catalyst mesh size decreased (entry 3 vs 7–8), indicating that this parameter plays a crucial role in the transformation.

After determining the appropriate catalyst loading and mesh size, studies to determine the best type of solvent (entries 9–11) and base (entries 12–18) for this transformation were carried out. With regard to the solvent, DMSO was found to provide the best results (entry 3 vs 9–11) and of the bases tested, $\hbox {K}_{2}\hbox {CO}_{3}$ resulted in the highest yield (entry 3 vs 13–16). There was no reaction when the base was removed (entry 12), demonstrating the importance of the presence of a base in the reaction. After selecting the base, its stoichiometry was also investigated (entries 17–18) and varying the quantity of base had a considerable effect (entry 3 vs 17–18).

The reaction demonstrated sensitivity to the mesh size of selenium similar to that of the catalyst mesh size. When elemental selenium with a larger mesh size was used, there was a sharp decrease in the yield of **3a** (entry 3 vs 19–20). The reaction time and temperature were then evaluated for this selenylation reaction (entries 21–24) and optimum values of $120\ ^{\circ }\hbox {C}$ and 12 h were identified.

**Scheme 1:**
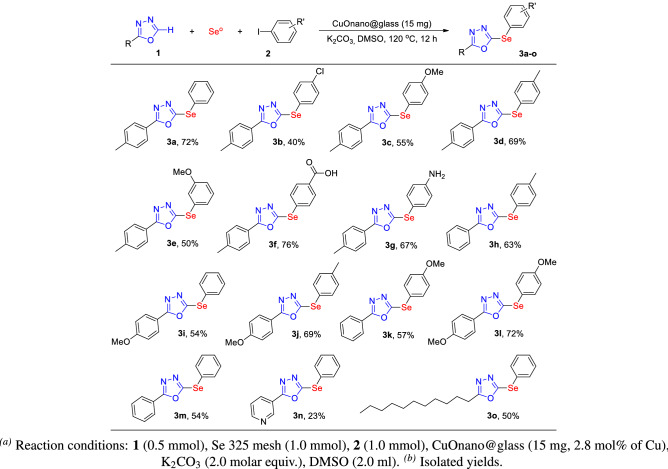
Scope of oxadiazole 1 and aryl iodides $2.^{a,b}$.

Having determined the best conditions (Table S1, entry 3), they were applied to evaluate the generality and scope of the selenylation reaction (Scheme 1). The methodology presented good yields when electron withdrawing (**3b**) and donating (**3c–e**) groups were present in the iodoarene, and different functional groups, such as carboxylic acid (**3f**) and aniline (**3g**), were tolerated, generating the selenylated product with good yields.

Modifications to the ODZ moiety did not have a negative effect on the reaction, with yields similar to that of **3a** (**3h–m**). Selenylated products with heteroarene and alkyl functionalized oxadiazoles were also successfully obtained (**3n** and **3o**).

**Scheme 2:**
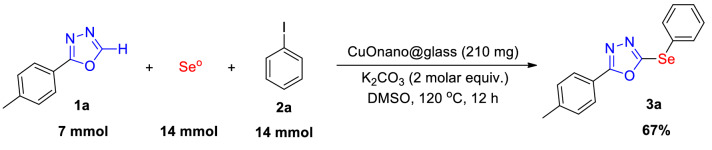
Gram-scale reaction.

In order to demonstrate the synthetic utility of this protocol, a gram-scale (7.0 mmol) reaction was carried out (Scheme 2). In this case, oxadiazole **1a** and iodobenzene **2a** were selected as the reagents and the optimized conditions were applied, affording the selenylated product **3a** with 67% yield. This protocol thus represents a practical method for the preparation of biologically-relevant compounds.

**Scheme 3:**
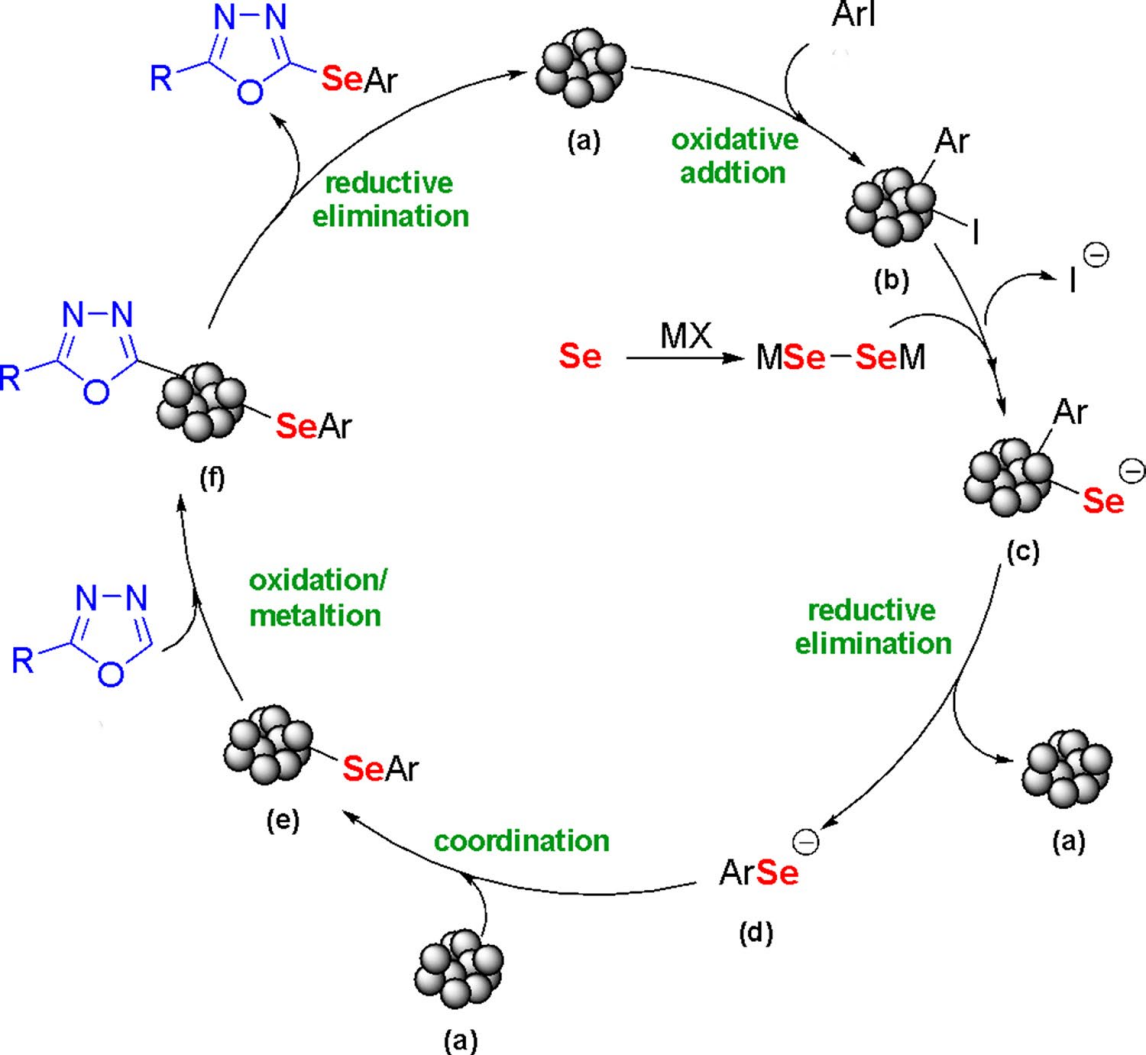
Plausible proposal for the reaction mechanism.

Based on previous reports^27–29^, a plausible mechanism can be proposed (Scheme 3). In the presence of base, selenium generated selenolate or diselenolate anions^48,49^. The catalytic cycle could begin from the formation of complex **(b)** from the CuOnano@glass catalyst **(a)** via oxidative addition. Ligand exchange could then occur with the diselenolate anion, leading to complex **(c)**. In the next step, complex **(c)** could undergo reductive elimination to afford aryl-selenolate **(d)**, which, on coordination with the catalyst **(a)**, results in species **(e)**. The subsequent C–H bond metalation of oxadiazole might result in species **(f)** followed by the immediate reductive elimination to afford the selenylated product and regenerate the catalyst **(a)**.

### Phenol hydroxylation

Notwithstanding the lower reactivity associated to the silica-based glasses, the use of glasses, in particular phosphate-based, as catalyst materials enables an unparalleled opportunity for catalysts development. Borophosphate glasses can be easily doped with transition metals by dissolving oxides/nitrates in the glass melt. The annealing under a controlled atmosphere provides an unusual approach for the synthesis of immobilized silver^2^, copper^4^, and nickel^32^, $$^{(\mathrm{b})}$$ nanostructures. Thus, the glass can act as an active medium for the growth and support of transition metal nanoparticles in a single step.

**Figure 5:**
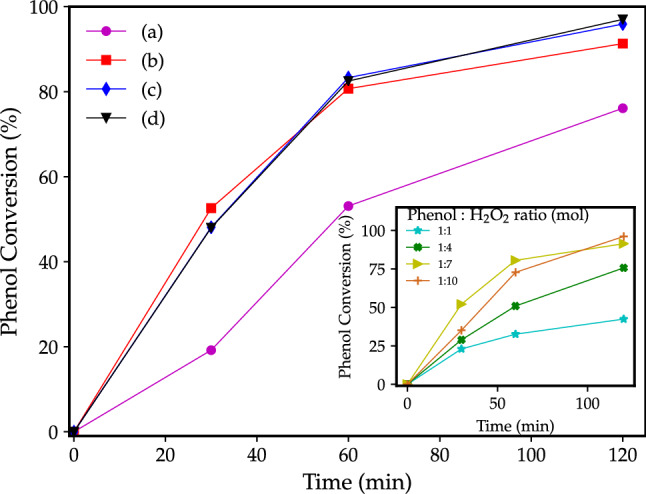
Effect CuOnano@glass catalyst at copper wt% (glass mass) on the phenol hydroxylation reaction at $50\ ^{\circ }\hbox {C}$ (a) 1.09 mg (25 mg ), (b) 2.17 (50 mg), (c) 3.26 mg (75 mg), and (d) 4.34 (100 mg). Graph in insert: effect of phenol: $\hbox {H}_{2}\hbox {O}_{2}$ ratio in the catalytic reaction.

The aromatic hydroxylation can be accomplished in the presence of $\hbox {H}_{2}\hbox {O}_{2}$ and a catalyst. The CuOnano@glass catalyst promotes the formation of $\hbox {OH}^{.}$ radicals that oxidize the phenol. The graph in insert (Fig. 5) shows the effect of Phenol: $\hbox {H}_{2}\hbox {O}_{2}$ ratio on the catalyses. The increase of $\hbox {H}_{2}\hbox {O}_{2}$ content rise rapidly the conversion from 42.4 to 96.1% for phenol:$\hbox {H}_{2}\hbox {O}_{2}$ ratio 1:1 to 1:10, respectively. Despite the increase of phenol:$\hbox {H}_{2}\hbox {O}_{2}$ ratio from 1:7 to 1:10, the phenol conversion increase only from 91.4 to 96.1%. The main image (Fig. 5) shows the effect of the 6 mol% (theoretical) copper-doped (experimental 6.4 mol% or 4.34 wt% of copper) glass catalyst mass on the phenol hydroxylation. The reactions were performed with a glass catalyst loading of 100, 75, 50, and 25 mg which results in a copper amount of 4.34, 3.26, 2.17, and 1.09 mg, respectively. Although it is expected an increase of the reaction effectiveness with the catalyst amount, the reaction shows a slight improvement with further additions of catalyst from 50 mg. Furthermore, the reactions performed with 50, 75, and 100 mg of copper glass catalyst show an identical performance for the reaction times, reaching a phenol conversion of 97% with the addition of 100 mg of the glass-based catalyst (Fig. 5d). No phenol hidroxilation was noticed using the undoped glass after 120 min and extended reaction time of 180 min. At least, Fig. S1 (Electronic Supplementary Information) shows the effect of glass particle size (increase in the Mesh sieve) on the phenol hydroxylation. The decrease in particle size from 200–250 to 250–325 Mesh range results in a slight increase of conversion from 92.4 to 93.4% ($<1 \%$), while the decrease to 325–400 Mesh reaches 98.2% of conversion, an increase of 5%. The conversion rise with the decrease of the glass particle size (lower limit) from $63\ \mu \hbox {m}$ (250 Mesh) to $38\ \mu \hbox {m}$ (400 Mesh). The fragile nature of glass materials enables to obtain reduced particle sizes. Thus, the 325–400 Mesh range can be easily used for catalyst development.

### Reduction of 4-nitrophenol

**Figure 6:**
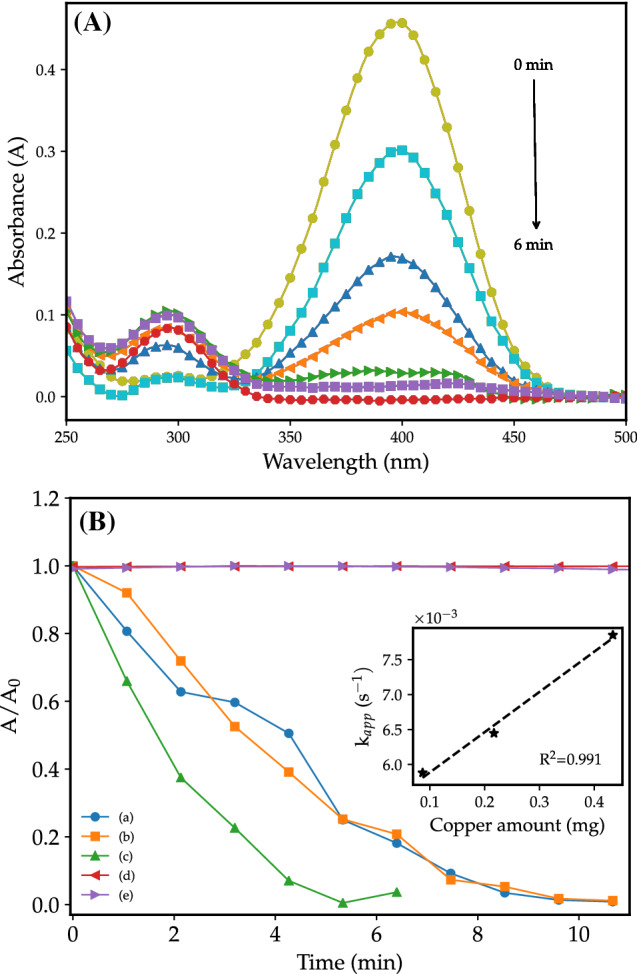
(**A**) Typical UV–Vis absorption spectra for 4-nitrophenol reduction by CuOnano@glass catalyst with 0.43 mg of copper (10 mg of glass) and (**B**) Effect of CuOnano@glass catalyst mass at copper wt% (glass mass) on normalized absorbance ($\hbox {A}/\hbox {A}_0$) versus time (min) for (a) 0.09 mg (2 mg), (b) 0.22 mg (5 mg), (c) 0.43 mg (10 mg), (d) Undoped borophosphate glass, and (e) Without catalyst. Graphic in insert: evolution of $\hbox {k}_{app}$ as a function of copper amount.

The successful application of the catalyst on organic synthesis and an oxidation process (phenol hydroxylation) encourages the use of the CuOnano@glass in an opposite scenario. In this sense, the reduction of the 4-nitrophenol was used as a model reaction. The 4-enolate at the presence of a metallic catalyst and hydride ions (H–) leads to the 4-aminophenol. The reaction progress can be monitored by changes in the initial absorbance ($\hbox {A}_0$) at 400 nm over time^50,51^.

Figure 6A shows a typical reaction using CuOnano@glass catalyst for 4-nitrophenol reduction by $\hbox {NaBH}_{4}$. The thermal treatment under air atmosphere results in the oxidation of the metallic copper nanoparticles and the formation of CuO nanoparticles (Fig. 1). However, the reduction proceeds by the transference of surface-hydrogen species adsorbed on the surface of metallic nanostructures to the 4-nitroenolate^51^. The immersion of CuOnano@glass in a $\hbox {NaBH}_{4}$ solution results in gas evolution just after the addition (Fig. S1 video at Supplementary Electronic Information). The reaction of hydride ions (H–) with CuO oxide supported on the glass surface form an in situ fresh copper metallic layer. Usually, the apparent reaction rate ($\hbox {k}_{app}$) is interpreted as a measure of the catalytic activity^50^. However, $\hbox {k}_{app}$ shows a dependency on the amount of the catalyst (Graphic in insert Fig. 6). Similarly, the increase in the amount of copper ions in the glass matrix results in a $\hbox {k}_{app}$ increase with the amount of copper in the glass matrix from 3 to 6% of copper. Further increase, from 9 to 12%, results in a slight increment in the $\hbox {k}_{app}$ value (Fig. S3, Electronic Supplementary Information). We associate the increase in the size and/or the coalescence of copper-based particles leading to a reduction in their surface areas, so does the catalytic activity. To evaluate the stability of the CuOnano@glass catalyst, a typical 4-nitrophenol reaction was performed and the mass of the catalyst was employed in a reuse test. After the reactions, the catalyst was cleaned with water followed by ethanol (3 times), and dried at room temperature under air atmosphere. After 5 days, the catalyst was used in a second run, resulting in a conversion of 98 % for CuOnano@glass catalyst doped with 6 mol% of copper ions (Fig. S4, Electronic Supplementary Information).

The catalytic effectiveness should be measured on the basis of activity parameter ($\kappa$) which can be mass normalized ($\kappa _m$ ($\text {s}^{-1}\cdot \text {g}^{-1}$))^31,50,52^. The apparent reaction rate can be obtained by the slope of the plot $\ln$($\hbox {A}/\hbox {A}_0$) against time. Table 1 summarizes the apparent reaction rate ($\hbox {k}_{app}$), the activity parameter ($\kappa _m$), and provides a comparison among those values for some reported copper-based catalysts. The CuOnano@glass catalyst shows higher $\kappa _m$ than reported copper-based catalysts ($\hbox {Cu}_{0}$, $\hbox {Cu}_{2}\hbox {O}$ and/or Cu). Yang et al.^53^ reported an outstanding metal-based phosphate with a reconstruction of reactive active sites with Cu and Ni/Cu catalyst. It is worth mentioning that CuOnano@glass catalyst synthesis is solvent-free and carried out using the widespread melting-quenching technique which enables glass synthesis at a large scale with simple raw chemicals.

**Table 1:** Catalytic effectiveness parameters for reported catalysts.

Catalyst	Mass^d^	$\hbox {k}_{app}(10^{-3})$	$\kappa _m$	Ref.
	mg	$\hbox {s}^{-1}$	$\hbox {s}^{-1} \cdot \hbox {g}^{-1}$	
CuOnano@glass	0.09	5.88	**67**.**7**	Present work
	0.22	6.45	29.7	
	0.43	7.85	18.1	
Cu-P-RT^a^	2.0	62.0	31.0	^53^
Ni/Cu-P-RT^a^	2.0	70.5	35.3	^53^
$\hbox {Cu}_{2}\hbox {O}$–Cu–CuO	0.5	10.4	20.7	^52^
Cu/carbon^b^	1.4	6.56	4.7	^54^
CD/CuO/mHA^c^	1.1	4.23	3.8	^55^
CuO@$\hbox {Ag}^0$	5.0	1.97	0.4	^56^
CuO	5.0	1.37	0.3	^56^

^a^Crystalline mono and bimetallic (Cu and Ni) phosphate.

^b^Catalyst 0.5 g/L, Volume 3.1 mL, and Cu detected 92%.

^c^Carbon dots (CD)/CuO supported on mesoporous hydroxyapatite (mHA), catalyst 10 mg—11 wt% of Cu.

^d^Cu present in the CuOnano@glass catalyst.

## Conclusions

In conclusion, supported copper oxide nanoparticles obtained by annealing copper-doped borophosphate glass were evaluated as a catalyst. The annealing of borophosphate glass under a reductive atmosphere enables the growth of metallic nanoparticles on the glass surface. Additional annealing, under an air atmosphere, oxidizes the metallic nanostructures and supported cupric oxide forms on the glass surface. The glass-based CuONPs presented outstanding performance as catalyst in three different types of useful organic transformations. It could act as catalyst in the hydroxylation of phenol as well as the reduction of 4-nitrophenol. In addition, these materials were found to be an efficient nano-catalyst in a three-component reaction using structurally diverse 1,3,4-oxadiaozles, elemental selenium and aryl iodides, resulting in selenylated oxadiazoles via C($\hbox {sp}^2$)-H bond selenylation. The important features of this benign protocol are: (1) reaction performed open to the air; (2) atom-economic; (3) inexpensive; (4) non-toxic and safe catalytic system; (5) low catalyst loading; (6) gram-scalable; and (7) applicable to structurally-diverse oxadiazoles and aryl iodides. The synthetic application described herein represents an alternative and eco-friendly approach for the preparation of selenylated oxadiazole which has therapeutic potential.

## Supplementary information

Supplementary Information.

Supplementary Figure S2.
