# Supplementary material for: Borophosphate glass as an active media for CuO nanoparticle growth: an efficient catalyst for selenylation of oxadiazoles and application in redox reactions
Source: Sci Rep. 2020 Sep 17;10:15233. doi: 10.1038/s41598-020-72129-w (PMC7498614; doi:10.1038/s41598-020-72129-w)
Supplement: Supplementary file 1 — Supplementary Information. [file 41598_2020_72129_MOESM1_ESM.pdf]

Electronic Supplementary Information: Borophosphate glass as an active media for CuO nanoparticle growth: an efficient catalyst for selenylation of oxadiazoles and application in redox reactions

Marcos R. Scheide[1], Marcos M. Peterle[1,+], Sumbal Saba[2,+], José S. S. Neto[1,+], Guilherme F. Lenz[3,+], Rosane Dias Cezar[4,+], Jorlandio F. Felix[5], Giancarlo V. Botteselle[6,‡], Ricardo Schneider[7,§], Jamal Rafique[3,*], and Antonio L. Braga[1,†]

[1]Universidade Federal de Santa Cararina - UFSC, Departamento de Química, 88040-900, Florianópolis SC, Brazil
[2]Universidade Federal do ABC, Centro de Ciências Naturais e Humanas-CCNH, Santo André, 09210-580, SP, Brazil
[3]Universidade Federal do Paraná - UFPR, Departamento de Engenharias e Exatas, 85950-000, Palotina PR, Brazil
[4]Universidade Federal do Mato Grosso do Sul - UFMS, Instituto de Química, Campo Grande, 79074-460, MS-Brazil
[5]Universidade de Brasília - UNB, Instituto de Física, Núcleo de Física Aplicada, 70910-900, Brasília DF, Brazil
[6]Universidade Estadual do Oeste do Paraná - UNIOESTE, Centro de Engenharias e Ciências Exatas (CECE), 85903-000, Toledo PR, Brazil
[7]Universidade Tecnológica Federal do Paraná - UTFPR, Group of Polymers and Nanostructures, 85902-490, Toledo PR, Brazil
[‡]gian.botteselle@gmail.com
[§]rschneider@utfpr.edu.br
[*]jamal.chm@gmail.com
[†]braga.anotnio@ufsc.br

August 11, 2020

Table S 1. Optimization of the reaction conditions[a]

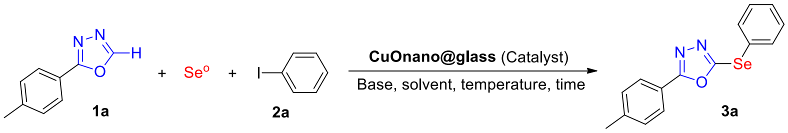

Entry	Catalyst mg	Base (eq.)	Solvent	T ℃	t h	Yield [b] %
1	10	K_2CO_3(2)	DMSO	120	12	48
2	12.5	K_2CO_3(2)	DMSO	120	12	68
3	15.0	K_2CO_3(2)	DMSO	120	12	72
4	17.5	K_2CO_3(2)	DMSO	120	12	60
5[c]	-	K_2CO_3(2)	DMSO	120	12	N.R.
6[d]	15.0	K_2CO_3(2)	DMSO	120	12	N.R.
7[e]	15.0	K_2CO_3(2)	DMSO	120	12	23
8[f]	15.0	K_2CO_3(2)	DMSO	120	12	27
9	15.0	K_2CO_3(2)	DMF	120	12	58
10	15.0	K_2CO_3(2)	Toluene	120	12	N.R.
11	15.0	K_2CO_3(2)	H_2O	120	12	N.R.
12	15.0	-	DMSO	120	12	N.R.
13	15.0	$KHCO_3$(2)	DMSO	120	12	70
14	15.0	Na_2CO_3(2)	DMSO	120	12	60
15	15.0	$NaHCO_3$(2)	DMSO	120	12	60
16	15.0	KOH(2)	DMSO	120	12	10
17	15.0	K_2CO_3(2.5)	DMSO	120	12	22
18	15.0	K_2CO_3(1)	DMSO	120	12	12
19[g]	15.0	K_2CO_3(2)	DMSO	120	12	52
20[h]	15.0	K_2CO_3(2)	DMSO	120	12	50
21	15.0	K_2CO_3(2)	DMSO	110	12	32
22	15.0	K_2CO_3(2)	DMSO	130	12	63
23	15.0	K_2CO_3(2)	DMSO	120	6	55
24	15.0	K_2CO_3(2)	DMSO	120	16	20

[a] Reaction conditions: **1a** (0.5 mmol), Se 325 mesh (1.0 mmol), **2a** (1.0 mmol), catalyst 325-400 Mesh (mg), base (molar equiv.), solvent (2.0 mL). [b] Isolated yields. [c] Without any catalyst. [d] Borophosphate glass without CuO. [e] Catalyst 250-325 Mesh. [f] Catalyst 200-250 Mesh. [g] Se 200 Mesh [h] Se 100 Mesh; N.R.= no reaction.

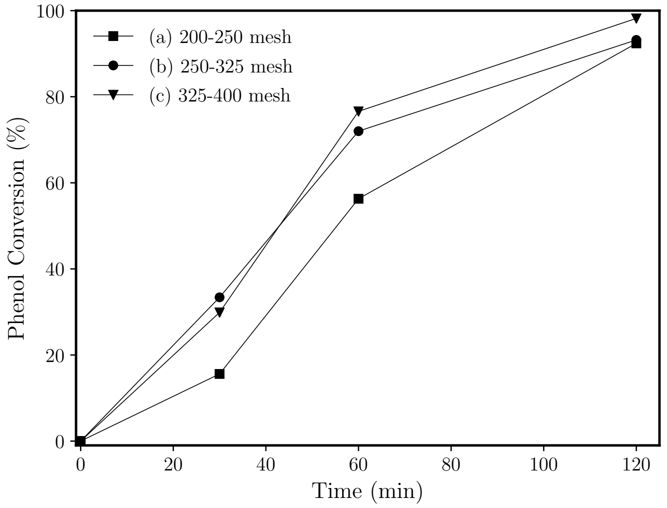

Figure S 1. Effect CuOnano@glass catalyst granulometry (Mesh) on phenol conversion. Experimental: phenol:H_2O_2 1:7, 3.26 mg copper weight (75 mg of glass).

test

Figure S 2. CuOnano@glass catalyst (5 mg) immersed in 1 mL of $NaBH_4$ solution. Please click on the image if the autorun does not work. *Note:* The video is available, in separate, as an ESI file as well.

A. Materials and Methods

Proton nuclear magnetic resonance spectra (^{1}H NMR) were obtained at 400 MHz on a Varian AS-400 NMR spectrometer. Spectra were recorded in $CDCl_3$ and DMSO-d_6 solutions. Chemical shifts are reported in ppm, referenced to the solvent peak of $CDCl_3$, DMSO-d_6 or tetramethylsilane (TMS) as the external reference. Data are reported as follows: chemical shift (δ), multiplicity, coupling constant (J) in Hertz and integrated intensity. Carbon-13 nuclear magnetic resonance spectra (^{13}C NMR) were obtained at 100 MHz on a Varian AS-400 NMR spectrometer. Spectra were recorded in $CDCl_3$ and DMSO-d_6 solutions. Chemical shifts are reported in ppm, referenced to the solvent peak of $CDCl_3$ and DMSO-d_6. Abbreviations to denote the multiplicity of a particular signal are: s (singlet), d (doublet), t (triplet), quint (quintet), dd (doublet of doublets), dt (doublet of triplets), ddd (doublet of doublet of doublets) and m (multiplet). High resolution mass spectra were recorded on a Bruker micrOTOF-Q II ESI, APPI or APCI mass spectrometer equipped with an automatic syringe pump for sample injection. The electron microscopy work has been performed with the JEM-1011 and JEOL JSM-6390LV microscopes of the LCME-UFSC for the TEM and SEM analysis, respectively. Infrared spectra were recorded on a Bruker Optics Alpha benchtop FT-IR spectrometer and are reported in frequency of absorption (cm^{-1}). The melting points were determined in a Microquimica MQRPF-301 digital model equipment with heating plate uncorrected. Column chromatography was performed using Silica Gel (230-400 mesh) following the methods described by Still. Thin layer chromatography (TLC) was performed using Merck Silica Gel GF254, 0.25 mm thickness. For visualization, TLC plates were either placed under ultraviolet light, or stained with iodine vapor and acidic vanillin. Most reactions were monitored by TLC for disappearance of starting material.

Unless otherwise stated, all reactions were carried out in open atmosphere; all reagents and solvents were obtained from commercial sources and used without any further purification. Oxadiazoles **2** were prepared according to the reported methods. [1,2] Reactions under inert atmosphere were conducted in flame-dried or oven dried glassware equipped with tightly fitted rubber septa and under a positive atmosphere of dry argon. Reagents and solvents were handled using standard syringe techniques. Temperatures were maintained by use of a mineral oil bath with an Ika heating and stirring plate.

For segregation of the catalyst, in a Falcon tube the reaction contents were added together with AcOEt (15 ml) and H_2O (5 mL). It was centrifuged for 6 min at 6000 rpm. The solution was removed and washed twice with AcOEt and the catalyst was dried in reduced pressure.

B. General Procedure for the synthesis of selenylated oxadiazoles:

In a Schlenck tube, containing DMSO (2 mL), the appropriate oxadiazole **1** (0.5 mmol), the respective iodo-(hetero)arene **2** (1.0 mmol), selenium 325 mesh (79.0 mg, 1.0 mmol), K_2CO_3 (138.2 mg, 1.0 mmol) and CuO/borophosphate glass (15.0 mg - 2,83 mol%) were added. The reaction was heated to 120 ºC in an oil bath for 12 h, with continuous stirring. After this, the mixture was diluted with ethyl acetate (15 mL) and washed with Brine (3 x 10 mL). The organic phase was separated, dried over $MgSO_4$ and concentrated under vacuum. The crude residue was purified by column chromatography utilizing silica gel as the stationary phase and eluted with a mixture of hexane/ethyl acetate.

C. Characterization data of compounds 3a-3o

2-(phenylselanyl)-5-(p-tolyl)-1,3,4-oxadiazole (3a)[3]

Obtained as a yellow solid; m.p: 58-60°C (lit. 65-66 ºC); Yield: 60%; Purified using hexane/ethyl acetate (95:5). **^{1}H NMR**(400 MHz, $CDCl_3$) δ 7.85 (d, *J* = 8.2 Hz, 2H), 7.77 (dt, *J* = 6.5, 1.6 Hz, 2H), 7.44 – 7.36 (m, 3H), 7.27 (d, *J* = 7.9 Hz, 2H), 2.40 (s, 3H). **^{13}C NMR** (100 MHz, $CDCl_3$) δ 167.5, 155.8, 142.5, 135.1, 129.9, 129.8, 129.7, 126.9, 124.5, 120.9, 21.8.

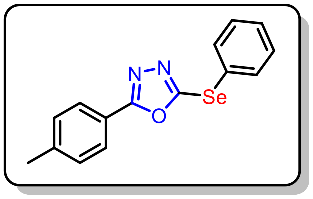

2-((4-chlorophenyl)selanyl)-5-(*p*-tolyl)-1,3,4-oxadiazole (3b)[4]

Obtained as a yellow solid; m.p: 87-88 ºC(lit. 82-83 ºC); Yield: 40%; Purified using hexane/ethyl acetate (95:5); **^{1}H NMR**(400 MHz, $CDCl_3$) δ 7.85 (d, *J* = 8.3 Hz, 2H), 7.71 (d, *J* = 8.5 Hz, 2H), 7.37 (d, *J* = 8.5 Hz, 2H), 7.28 (d, *J* = 8.0 Hz, 2H), 2.42 (s, 3H); **^{13}C NMR** (100 MHz, $CDCl_3$) δ 167.5, 155.3, 142.5, 136.3, 136.2, 130.2, 129.7, 126.8, 122.3, 120.6, 21.6.

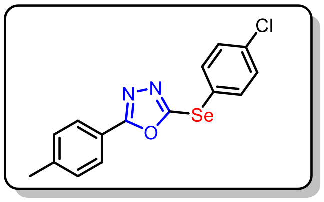

2-((4-methoxyphenyl)selanyl)-5-(p-tolyl)-1,3,4-oxadiazole (3c)[4]

Obtained as an off-white solid; m.p: 82-85°C (lit. 74-75 ºC); Yield: 55%; Purified using hexane/ethyl acetate (80:20). **^{1}H NMR** (400 MHz, $CDCl_3$) δ 7.82 (d, *J* = 8.1 Hz, 2H), 7.70 (d, *J* = 8.9 Hz, 2H), 7.25 (d, *J* = 8.2 Hz, 2H), 6.91 (d, *J* = 8.9 Hz, 2H), 3.81 (s, 3H), 2.39 (s, 3H). **^{13}C NMR**(100 MHz, $CDCl_3$) δ 167.3, 161.1, 156.62, 142.4, 137.6, 129.8, 126.8, 120.9, 115.6, 114.1, 55.5, 21.7.

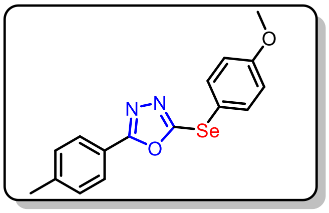

2-(p-tolyl)-5-(p-tolylselanyl)-1,3,4-oxadiazole (3d)[4]

Obtained as a white solid; m.p: 80-84°C (lit. 82-84 ºC); Purified using hexane/ethyl acetate (95:5); Yield: 69%; **^{1}H NMR** (400 MHz, $CDCl_3$) δ 7.82 (d, *J* = 8.3 Hz, 2H), 7.64 (d, *J* = 8.2 Hz, 2H), 7.24 (d, *J* = 8.5 Hz, 2H), 7.18 (d, *J* = 8.4 Hz, 2H), 2.38 (s, 3H), 2.35 (s, 3H). **^{13}C NMR** (100 MHz, $CDCl_3$) δ 167.2, 156.1, 142.3, 139.9, 135.2, 130.6, 129.7, 126.7, 120.8, 120.5, 21.6, 21.3.

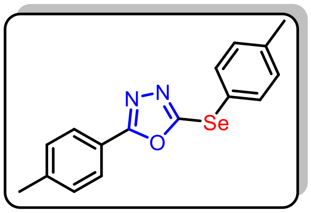

2-((3-methoxyphenyl)selanyl)-5-(p-tolyl)-1,3,4-oxadiazole (3e)[5]

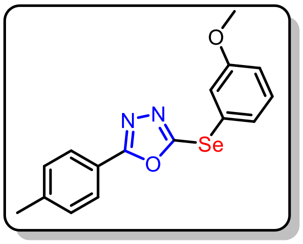

Obtained as a yellow oil; Yield: 50%; Purified using hexane/ethyl acetate (95:5). **^{1}H NMR** (400 MHz, $CDCl_3$) δ 7.85 (d, *J* = 8.2 Hz, 2H), 7.40 – 7.15 (m, 5H), 6.94 (ddd, *J* = 7.7, 2.6, 1.6 Hz, 1H), 3.80 (s, 3H), 2.39 (s, 3H). **^{13}C NMR** (100 MHz, $CDCl_3$) δ 167.4, 160.1, 155.6, 142.5, 130.5, 129.8, 126.9, 126.8, 125.1, 120.7, 119.9, 115.6, 55.5, 21.7.

4-((5-(p-tolyl)-1,3,4-oxadiazol-2-yl)selanyl)benzoic acid (3f)

At the end of the reaction, 5 mL of 1M aqueous solution of HCl was added to the yellow suspension followed by extraction; the compound was obtained as a dark brown solid; m.p: 117-120°C; Yield: 76%; Purified using hexane/ethyl acetate (80:20). **^{1}H NMR** (400 MHz, DMSO-d_6) δ 13.25 (s, 1H), 7.99 (d, *J* = 8.5 Hz, 2H), 7.89 – 7.82 (m, 4H), 7.39 (d, *J* = 8.0 Hz, 2H), 2.40 (s, 3H). **^{13}C NMR** (100 MHz, DMSO-d_6) δ 166.8, 166.7, 155.1, 142.4, 133.0, 131.7, 131.0, 130.4, 130.0, 126.5, 120.1, 21.16. **IR (solid, cm^{-1}) *ν*:** 3420; 2924; 1681; 1420; 1287; 1169; 583. **APPI-HMRS** *m/z* calculated for $C_{16}H_{13}N_2O_3Se$ $[M+H]^+$ 361.0087, found 361.0087.

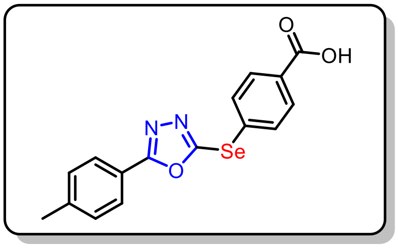

4-((5-(p-tolyl)-1,3,4-oxadiazol-2-yl)selanyl)aniline (3g)[5]

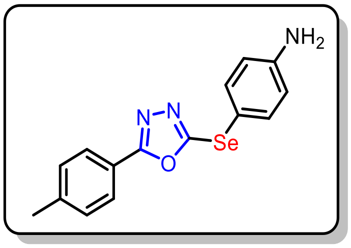

Obtained as a brown solid; m.p: 161-163 °C(lit. 166-168 °C); Yield: 67%; Purified using hexane/ethyl acetate (70:30). **^{1}H NMR** (400 MHz, $CDCl_3$) δ 7.82 (d, *J* = 8.3 Hz, 2H), 7.55 (d, *J* = 8.4 Hz, 2H), 7.25 (d, *J* = 8.8 Hz, 1H), 6.66 (d, *J* = 8.5 Hz, 2H), 3.96 (s, 2H), 2.40 (s, 3H). **^{13}C NMR** (100 MHz, $CDCl_3$) δ 167.1, 157.2, 148.4, 142.3, 137.7, 129.7, 126.8, 121.0, 116.1, 110.4, 21.7. **IR (solid, cm^{-1}) *ν*:** 3408; 3340; 3231; 1643; 1597; 1460; 1157; 831; 511. **APPI-HMRS** *m/z* calculated for $C_{15}H_{14}N_3Ose$ $[M+H]^+$ 332.0297, found 332.0296.

2-phenyl-5-(p-tolylselanyl)-1,3,4-oxadiazole (3h)

Obtained as a yellow solid; m.p:84-86ºC; Yield 63%; Purified using hexane/ethyl acetate (90:10). **^{1}H NMR** (400 MHz, $CDCl_3$) δ 7.95 (dd, *J* = 8.1, 1.5 Hz, 2H), 7.66 (d, *J* = 8.1 Hz, 2H), 7.51 – 7.43 (m, 3H), 7.20 (d, *J* = 7.9 Hz, 2H), 2.37 (s, 3H).**^{13}C NMR** (100 MHz, $CDCl_3$) δ 167.2, 156.7, 140.1, 135.4, 131.8, 130.7, 129.1, 126.9, 123.7, 120.50, 21.4. **IR (solid, cm^{-1}) *ν*:** 2924; 1544; 1464; 1135; 1664; 803; 713; 688; 490. **ESI-HRMS** *m/z* calculated for $C_{15}H_{13}N_2OSe$ $[M+H]^+$ 317.0188, found 317.0184.

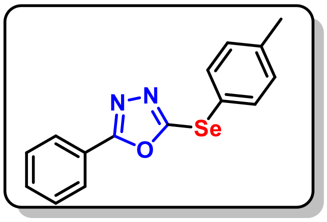

2-(4-methoxyphenyl)-5-(phenylselanyl)-1,3,4-oxadiazole (3i)[5]

Obtained as a yellow solid; m.p: 89-91 °C (lit. 89-90 ºC); Yield: 54%; Purified using hexane/ethyl acetate (90:10). **^{1}H NMR** (400 MHz, $CDCl_3$) δ 7.88 (d, *J* = 8.6 Hz, 2H), 7.75 (dd, *J* = 7.1, 1.1 Hz, 2H), 7.44 – 7.33 (m, 5H), 6.95 (d, *J* = 8.7 Hz, 2H), 3.83 (s, 3H). **^{13}C NMR** (100 MHz, $CDCl_3$) δ 167.2, 162.4, 155.3, 134.9, 129.8, 129.5, 128.6, 124.5, 116.0, 114.5, 55.5.

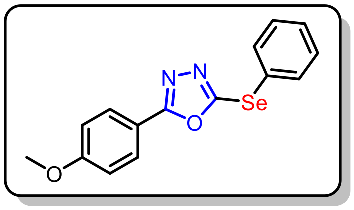

2-(4-methoxyphenyl)-5-(p-tolylselanyl)-1,3,4-oxadiazole (3j)

Obtained as an off-White solid; m.p: 91-92 ºC; Yield: 69%; Purified using hexane/ethyl acetate (90:10) as eluent. **^{1}H NMR** (400 MHz, $CDCl_3$) δ 7.88 (d, *J* = 8.9 Hz, 2H), 7.64 (d, *J* = 8.1 Hz, 2H), 7.18 (d, *J* = 7.9 Hz, 2H), 6.95 (d, *J* = 8.9 Hz, 2H), 3.84 (s, 3H), 2.36 (s, 3H). **^{13}C NMR** (101 MHz, $CDCl_3$) δ 167.1, 162.4, 155.7, 140.0, 135.2, 130.6, 128.6, 120.7, 116.1, 114.5, 55.5, 21.3. **IR (solid, cm^{-1}) *ν*:** 2921; 1609; 1495; 1262; 1020; 840; 490. **ESI-HRMS** *m/z* calculated for $C_{16}H_{15}N_2O_2Se$ $[M+H]^+$ 347.0294 found 347.0292.

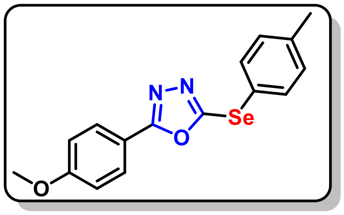

2-((4-methoxyphenyl)selanyl)-5-phenyl-1,3,4-oxadiazole (3k)

Obtained as a yellow solid; m.p: 79-80 ºC; Yield: 57%; Purified using hexane/ethyl acetate (90:10) as eluent. **^{1}H NMR** (400 MHz, $CDCl_3$) δ 7.93 (dd, *J* = 8.0, 1.4 Hz, 2H), 7.71 (d, *J* = 8.7 Hz, 2H), 7.58 – 7.31 (m, 3H), 6.92 (d, *J* = 8.8 Hz, 2H), 3.82 (s, 3H). **^{13}C NMR** (100 MHz, $CDCl_3$) δ 167.07, 161.11, 157.02, 137.57, 131.79, 129.05, 126.82, 123.67, 115.58, 113.94, 55.47. **IR (solid, cm^{-1}) *ν*:** 3057; 2942; 2837; 1584; 1544; 1488; 1454; 1253; 1023; 685; 515. **ESI-HRMS** *m/z* calculated for $C_{15}H_{13}N_2O_2Se$ $[M+H]^+$ 333.0137 found 333.0136.

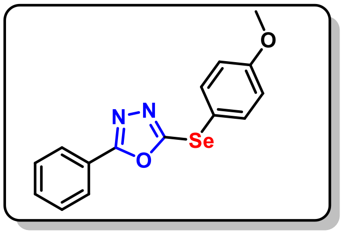

2-(4-methoxyphenyl)-5-((4-methoxyphenyl)selanyl)-1,3,4-oxadiazole (3l)

Obtained as an ff-white solid; m.p: 81-84ºC; Yield: 72%; Purified using hexane/ethyl acetate (85:15) as eluent. **^{1}H NMR** (400 MHz, $CDCl_3$) δ 7.87 (d, *J* = 9.0 Hz, 2H), 7.70 (d, *J* = 8.9 Hz, 2H), 6.95 (d, *J* = 9.0 Hz, 2H), 6.91 (d, *J* = 8.9 Hz, 2H), 3.84 (s, 3H), 3.81 (s, 3H). **^{13}C NMR** (100 MHz, $CDCl_3$) δ 167.0, 162.3, 161.0, 137.4, 128.6, 116.1, 115.5, 114.5, 114.1, 55.5, 55.4. **IR (solid, cm^{-1}) *ν*:** 2967; 1612; 1498; 1253; 1026; 828; 518. **APCI-HRMS** *m/z* calculated for $C_{16}H_{15}N_2O_3Se$ $[M+H]^+$ 363.02430, found 363.02432.

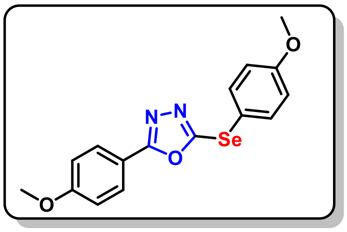

2-phenyl-5-(phenylselanyl)-1,3,4-oxadiazole (3m)[4]

Obtained as a yellow solid; m.p: 50-52°C (lit. 51-52 ºC); Yield: 54%; Purified using hexane/ethyl acetate (95:5). **^{1}H NMR** (400 MHz, $CDCl_3$) δ 7.95 (dt, J = 6.8, 1.5 Hz, 2H), 7.76 (dt, J = 6.5, 1.6 Hz, 2H), 7.54 – 7.34 (m, 6H). **^{13}C NMR** (100 MHz, $CDCl_3$) δ 167.2, 156.2, 135.1, 131.9, 129.9, 129.7, 129.1, 126.84, 124.3, 123.5.

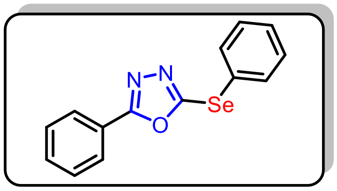

2-(phenylselanyl)-5-(pyridin-3-yl)-1,3,4-oxadiazole (3n)[5]

Obtained as a yellow solid; m.p: 86-88°C (lit. 87-88 ºC); Yield 23%; Purified using hexane/ethyl acetate (85:15). **^{1}H NMR** (400 MHz, $CDCl_3$) δ 9.16 (s, 1H), 8.83 – 8.70 (d, 1H), 8.26 (dt, J = 8.0, 1.9 Hz, 1H), 7.79 (dt, J = 6.7, 1.4 Hz, 2H), 7.50 – 7.37 (m, 4H). **^{13}C NMR** (101 MHz, $CDCl_3$) δ 165.1, 157.4, 152.5, 147.8, 135.5, 134.1, 130.0, 130.0, 123.9, 123.9, 123.8

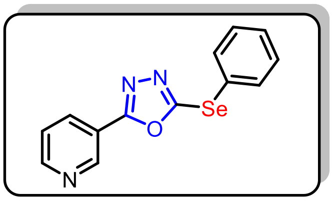

2-(phenylselanyl)-5-undecyl-1,3,4-oxadiazole (3o)[5]

Obtained as orange oil; Yield: 50%; Purified using hexane/ethyl acetate (95:5). **^{1}H NMR** (400 MHz, $CDCl_3$) δ 7.70 (d, J = 8.1 Hz, 2H), 7.43 – 7.33 (m, 3H), 2.80 (t, J = 7.6 Hz, 2H), 1.72 (q, J = 7.6 Hz, 2H), 1.25 (s, 16H), 0.88 (t, J = 6.9 Hz, 3H). **^{13}C NMR** (100 MHz, $CDCl_3$) δ 169.8, 134.9, 129.8, 129.5, 124.4, 31.9, 29.6, 29.6, 29.4, 29.3, 29.1, 28.9, 26.0, 25.4, 22.7, 14.2.

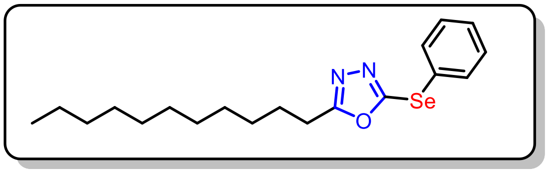

D. References

1. Kawano, T., Hirano, K., Satoh, T. & Miura, M. A new entry of amination reagents for heteroaromatic C-H bonds: Copper-catalyzed direct amination of azoles with chloroamines at room temperature. *J. Am. Chem. Soc.* **132**, 6900–6901 (2010).

2. Giles, D., Prakash, M. S. & Ramseshu, K. V. Synthesis and Biological Evaluation of Substituted Thiophenyl Derivatives of Indane-1 , 3-dione. *E-Journal Chem.* **4**, 428–433 (2007).

3. Hu, D., Liu, M., Wu, H., Gao, W. & Wu, G. Copper-catalyzed diarylation of Se with aryl iodides and heterocycles. *Org. Chem. Front.* **5**, 1352–1355 (2018).

4. Rafique, J., Saba, S., Rosário, A. R., Zeni, G. & Braga, A. L. K2CO3-mediated, direct C-H bond selenation and thiolation of 1,3,4-oxadiazoles in the absence of metal catalyst: An eco-friendly approach. *RSC Adv.* **4**, 51648–51652 (2014).

5. Peterle, M. M. *et al.* Copper-Catalyzed Three-Component Reaction of Oxadiazoles , Elemental Se / S and Aryl Iodides : Synthesis of Chalcogenyl (Se / S) -Oxadiazoles. *ChemistrySelect* **3**, 13191–13196 (2018).

E. NMR spectra of Compounds 3a-3o

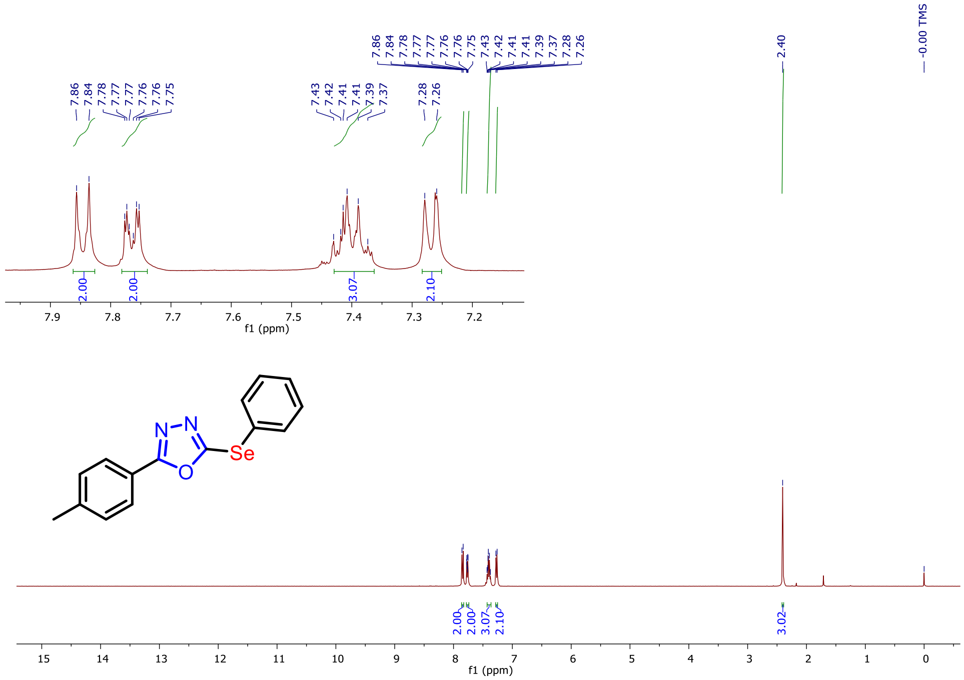

^{1}H NMR (400 MHz, $CDCl_3$) spectrum of 3a.

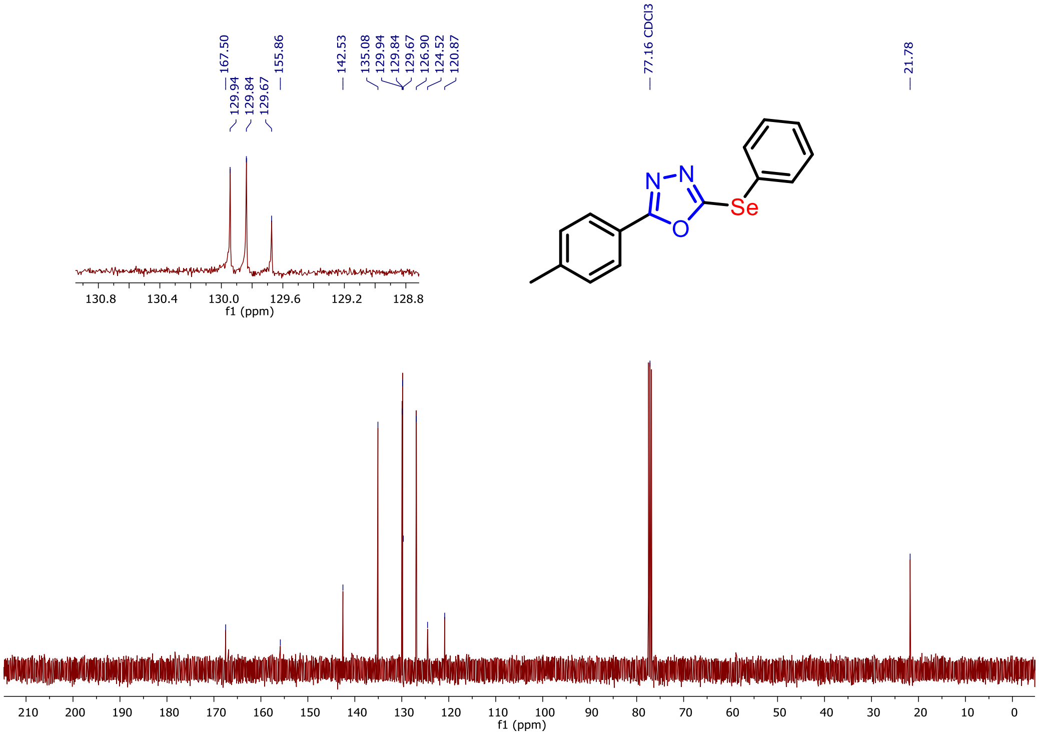

^{13}C NMR (100 MHz, $CDCl_3$) spectrum of 3a.

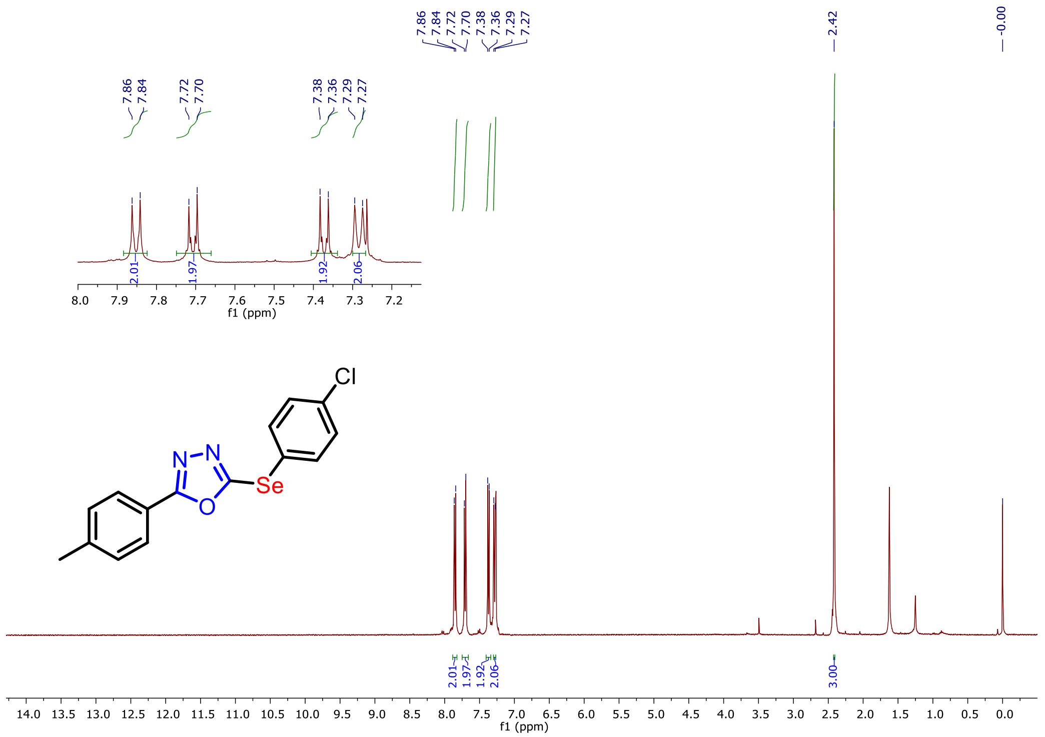

^{1}H NMR (400 MHz, $CDCl_3$) spectrum of 3b.

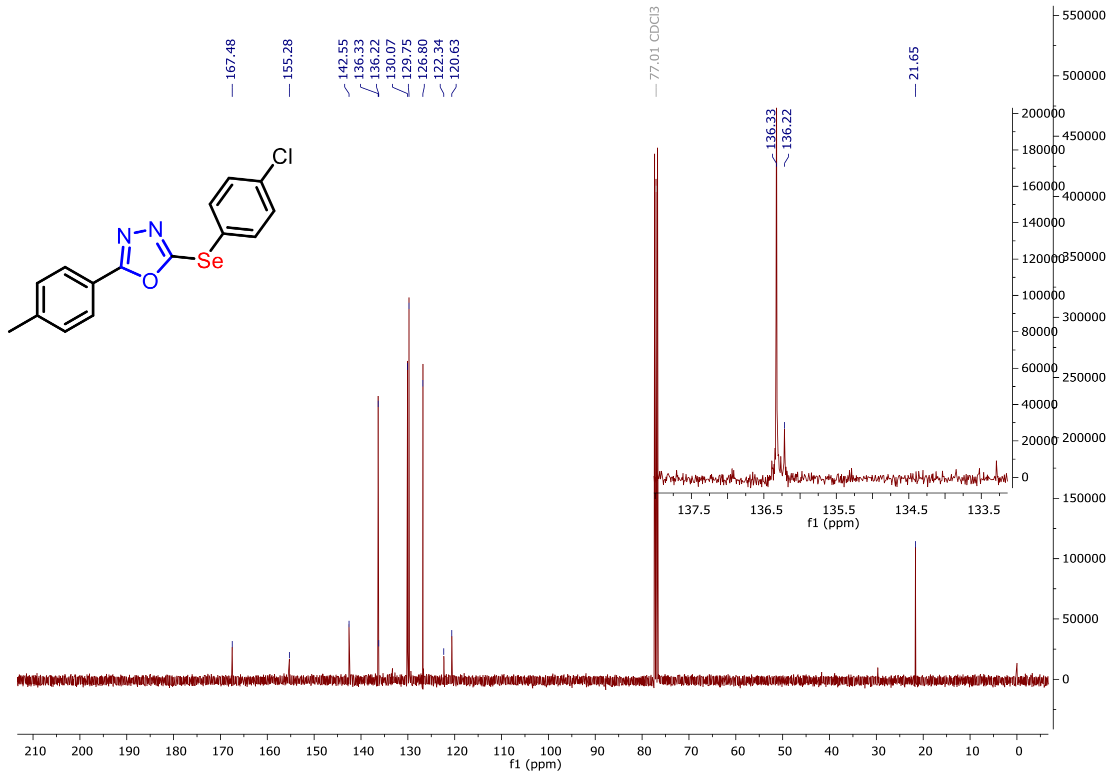

^{13}C NMR (100 MHz, $CDCl_3$) spectrum of 3b.

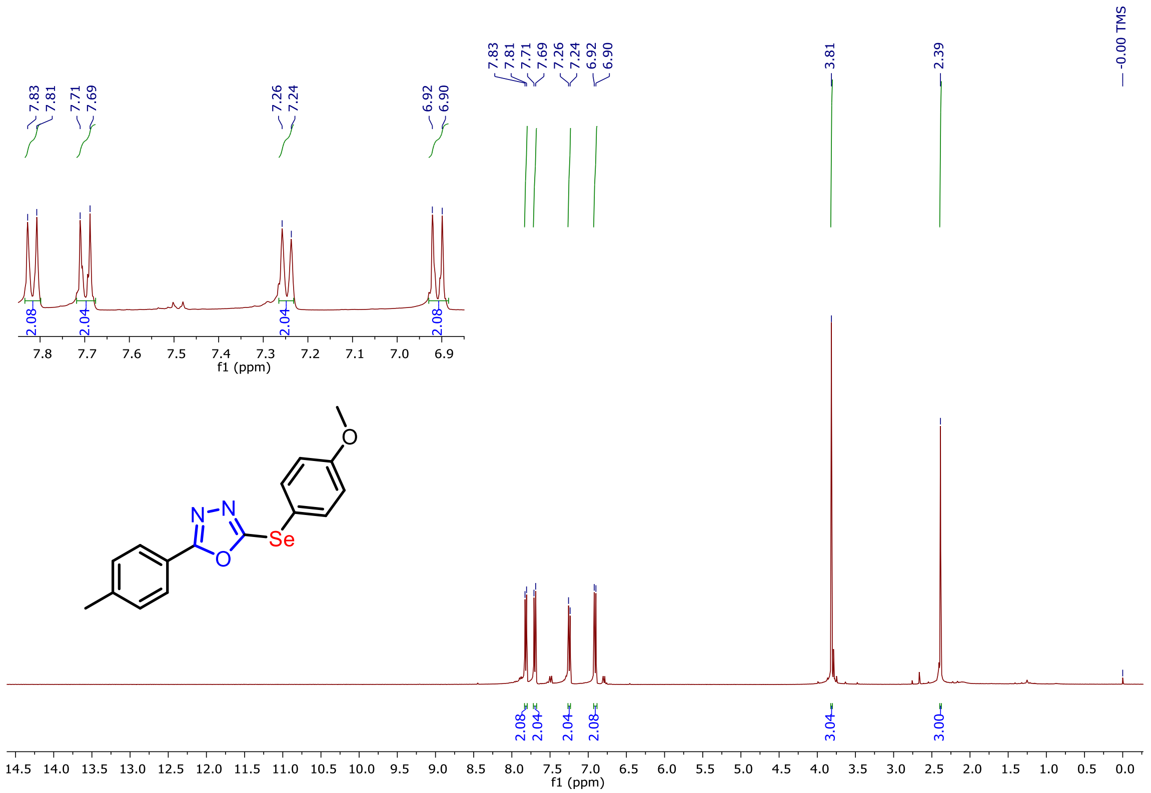

^{1}H NMR (400 MHz, $CDCl_3$) spectrum of 3c.

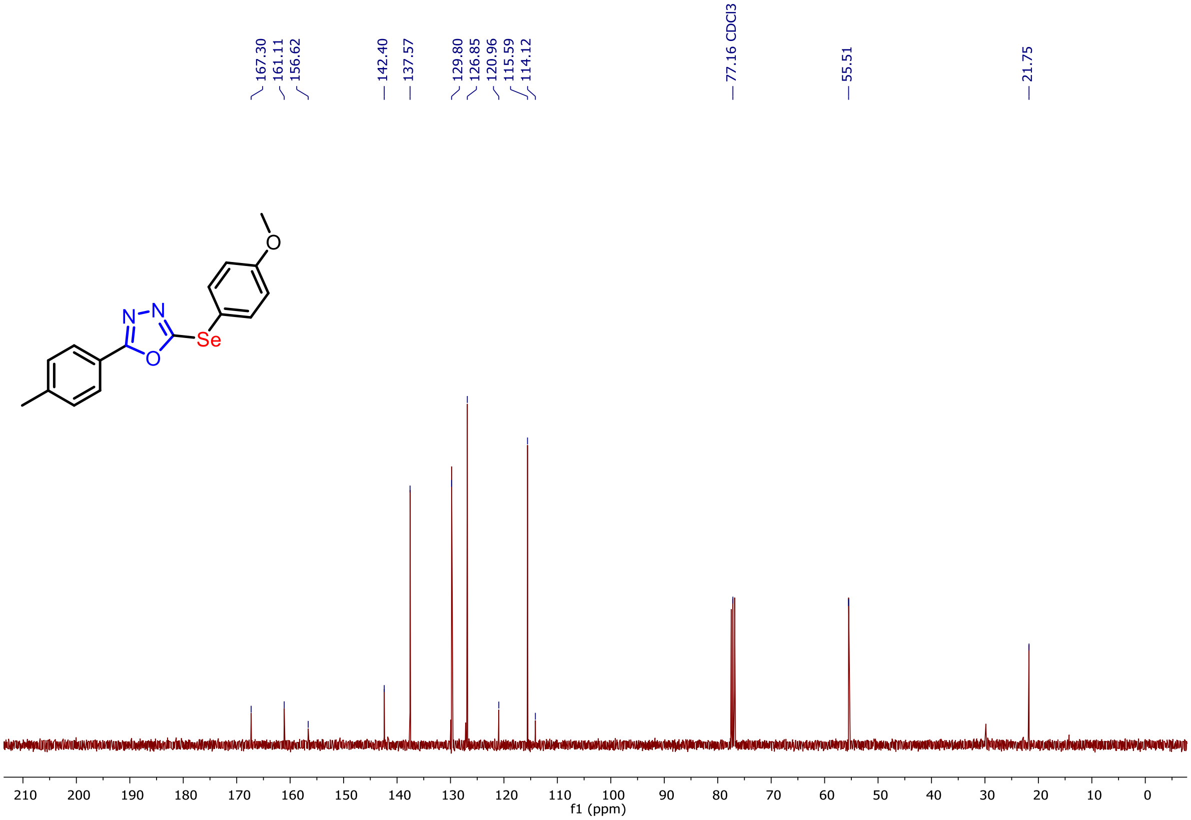

^{13}C NMR (100 MHz, $CDCl_3$) spectrum of 3c.

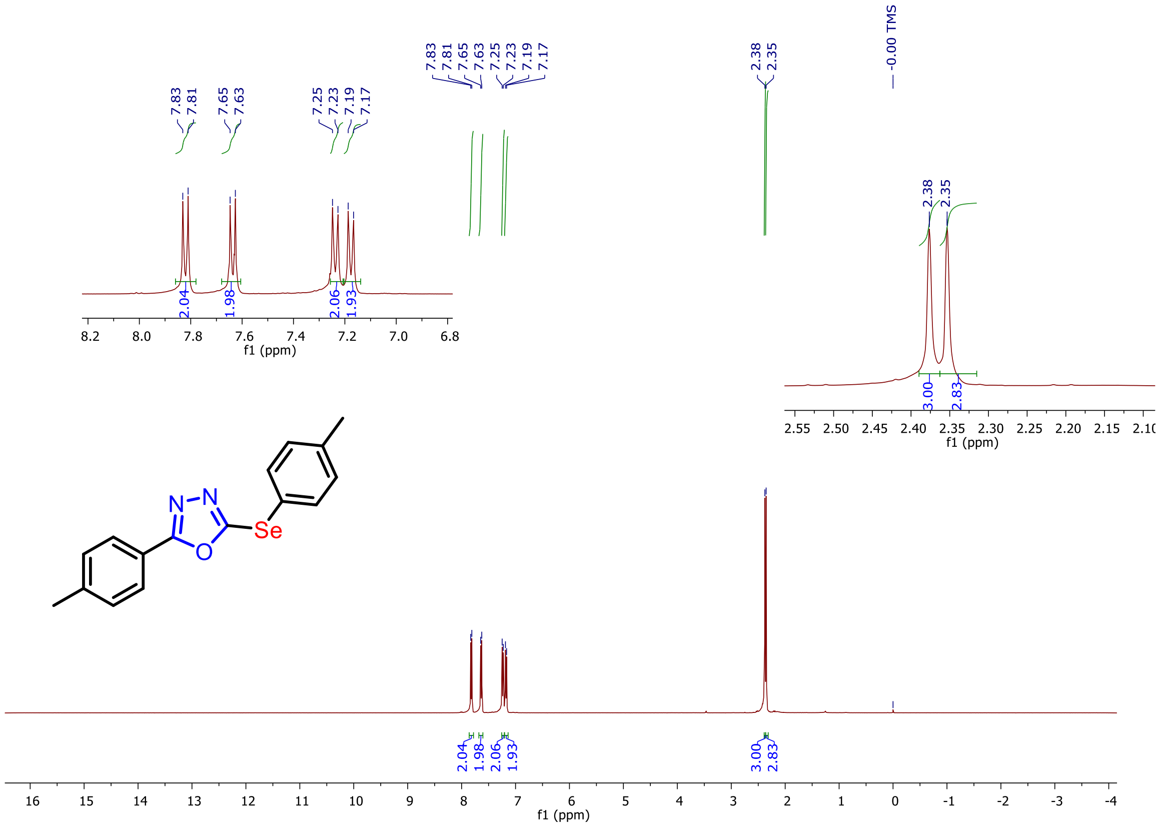

^{1}H NMR (400 MHz, $CDCl_3$) spectrum of 3d.

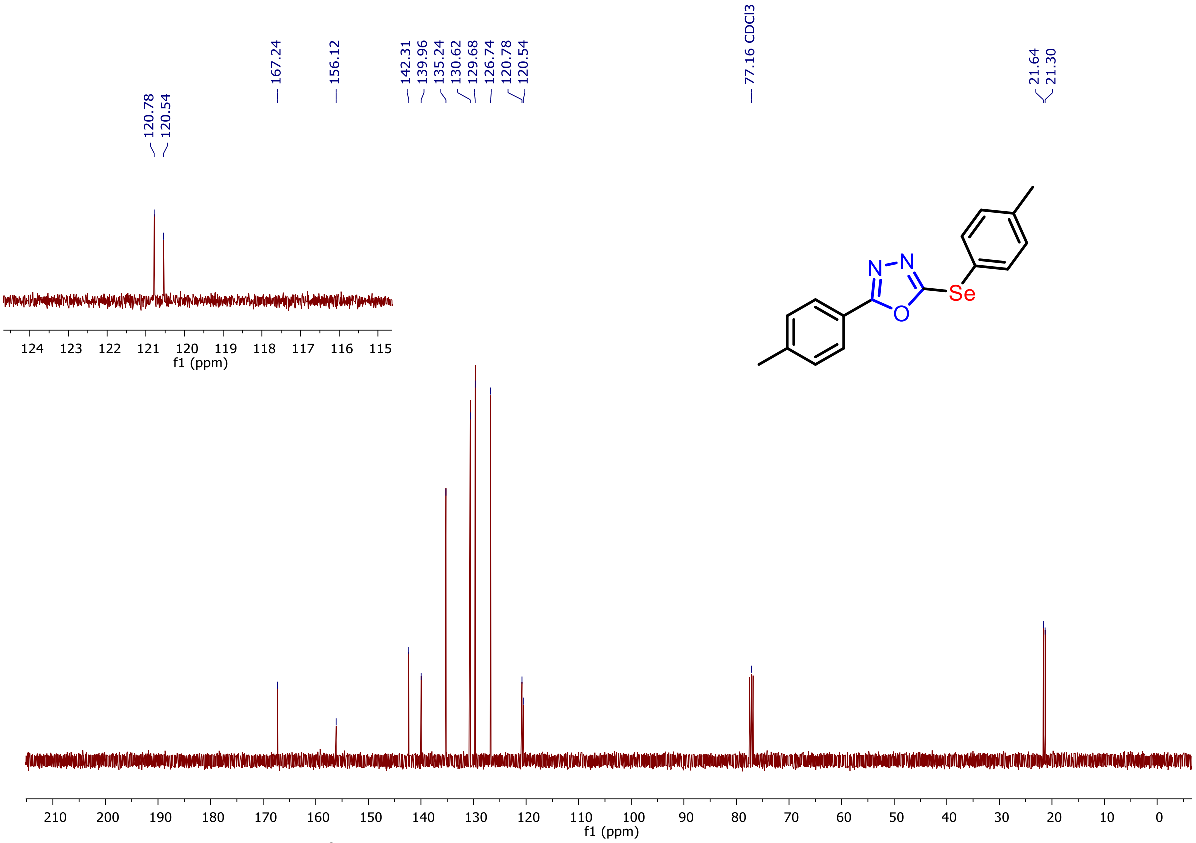

^{13}C NMR (100 MHz, $CDCl_3$) spectrum of 3d.

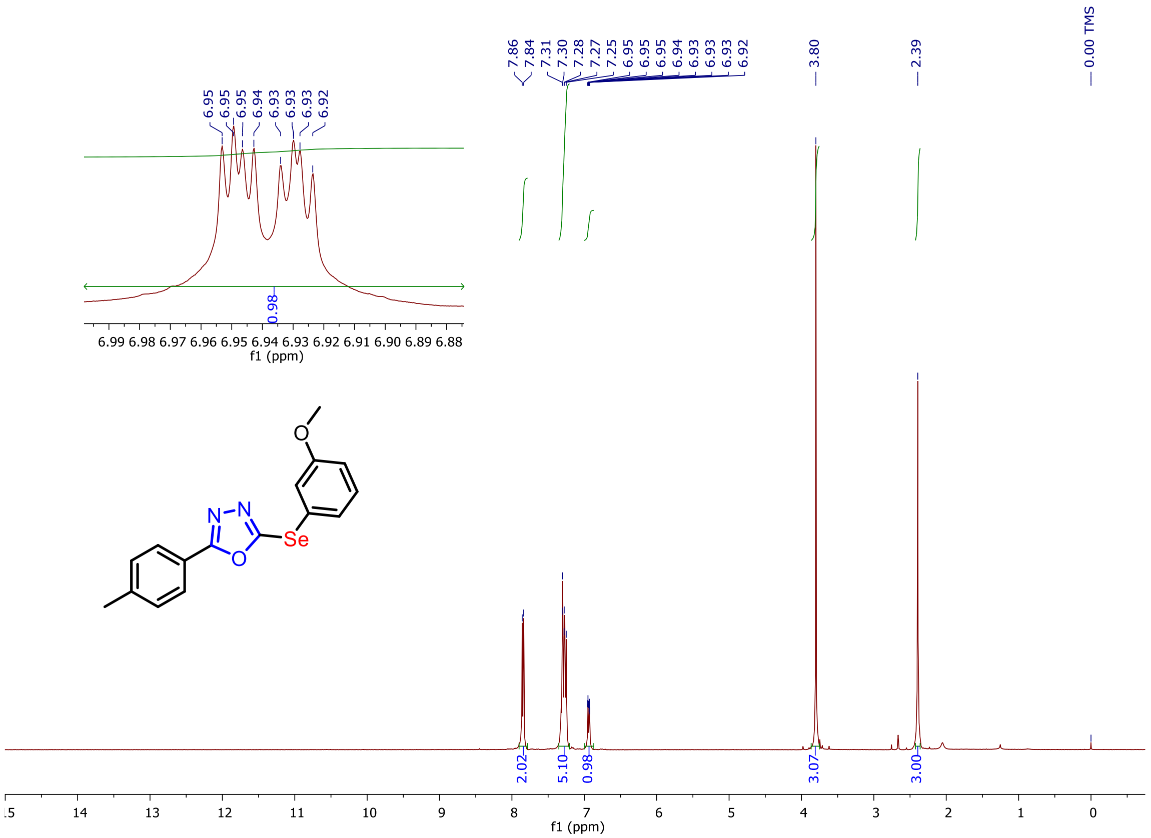

¹H NMR (400 MHz, $CDCl_3$) spectrum of 3e.

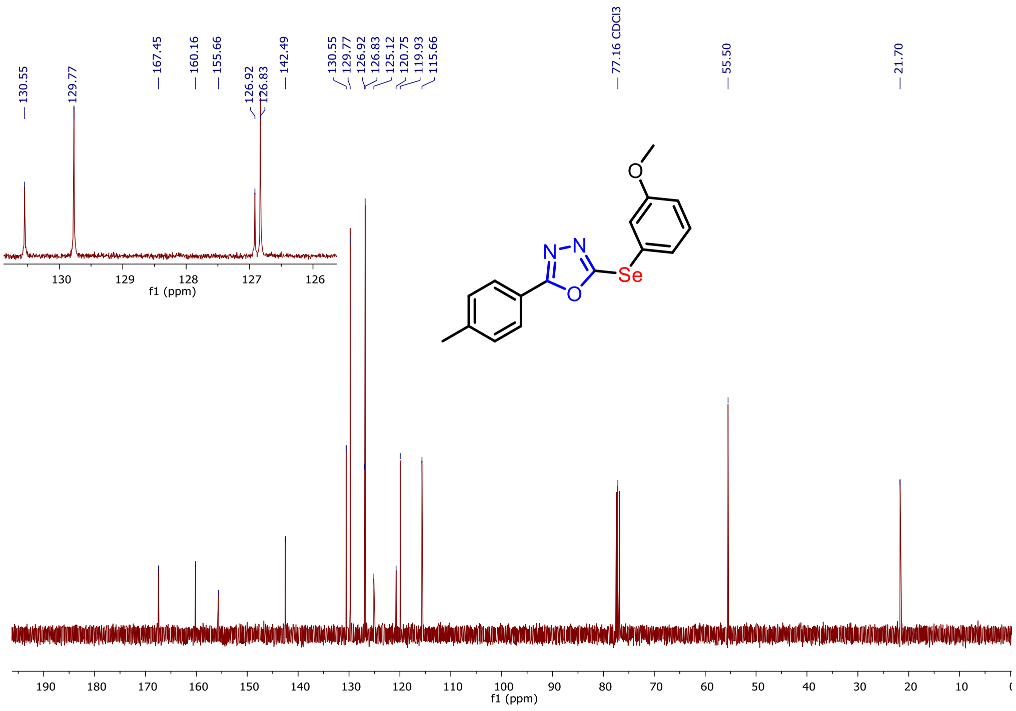

¹³C NMR (100 MHz, $CDCl_3$) spectrum of 3e

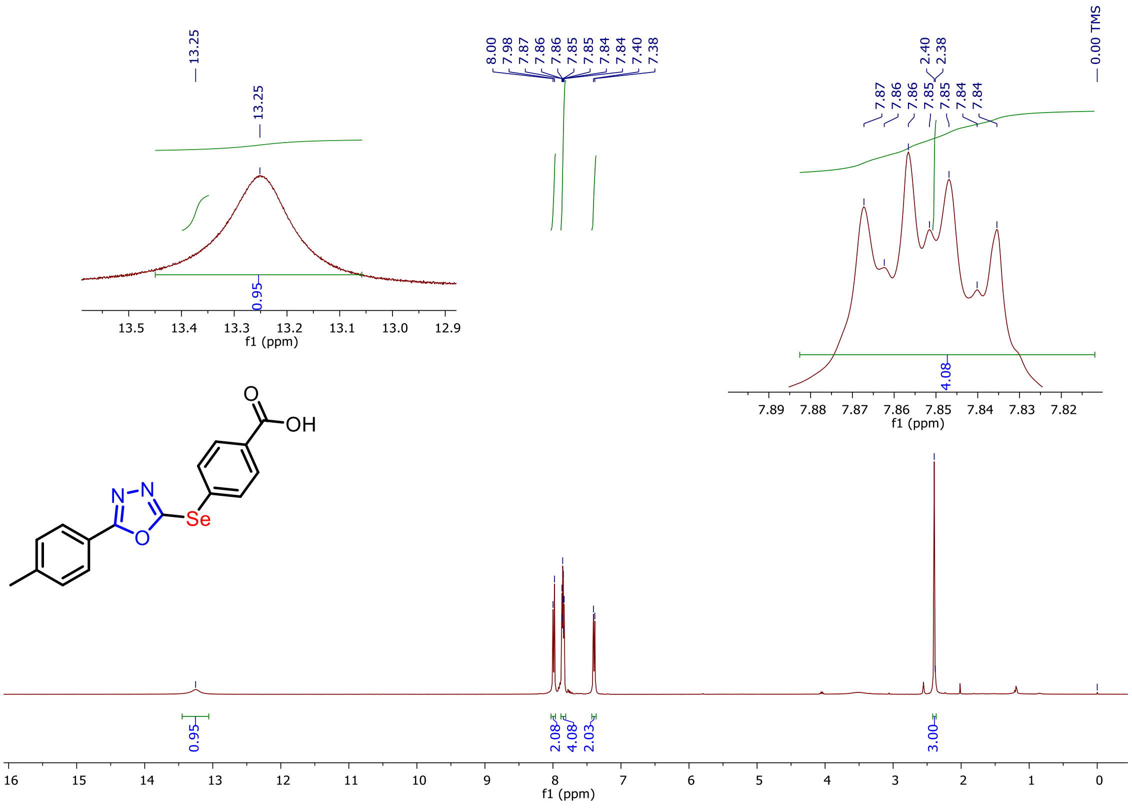

^{1}H NMR (400 MHz, DMSO-d_6) spectrum of 3f.

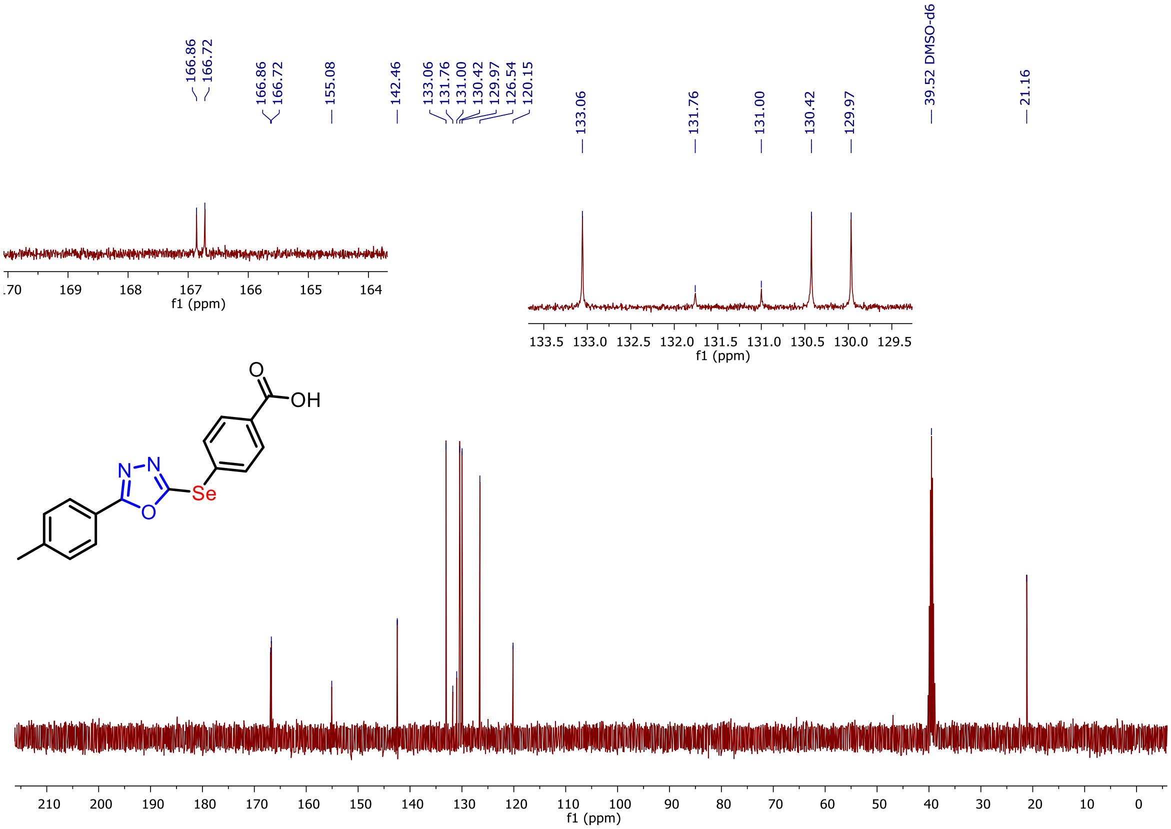

^{13}C NMR (100 MHz, DMSO-d_6) spectrum of 3f.

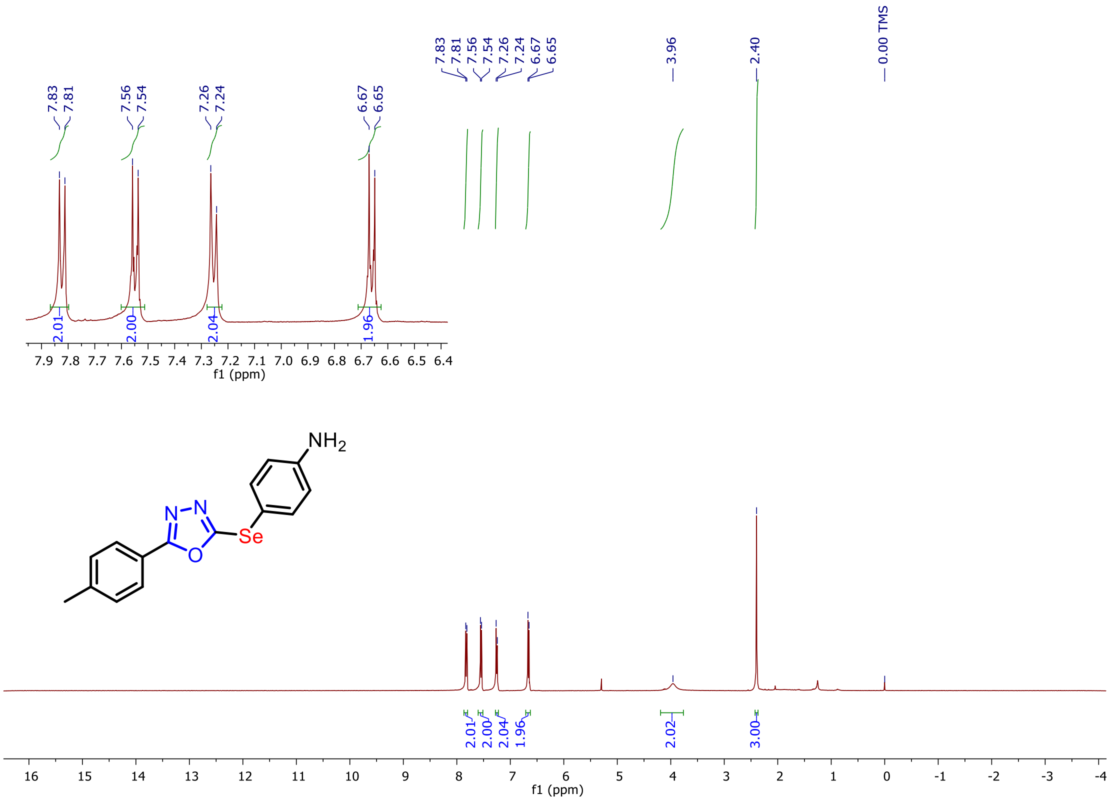

^{1}H NMR (400 MHz, $CDCl_3$) spectrum of 3g.

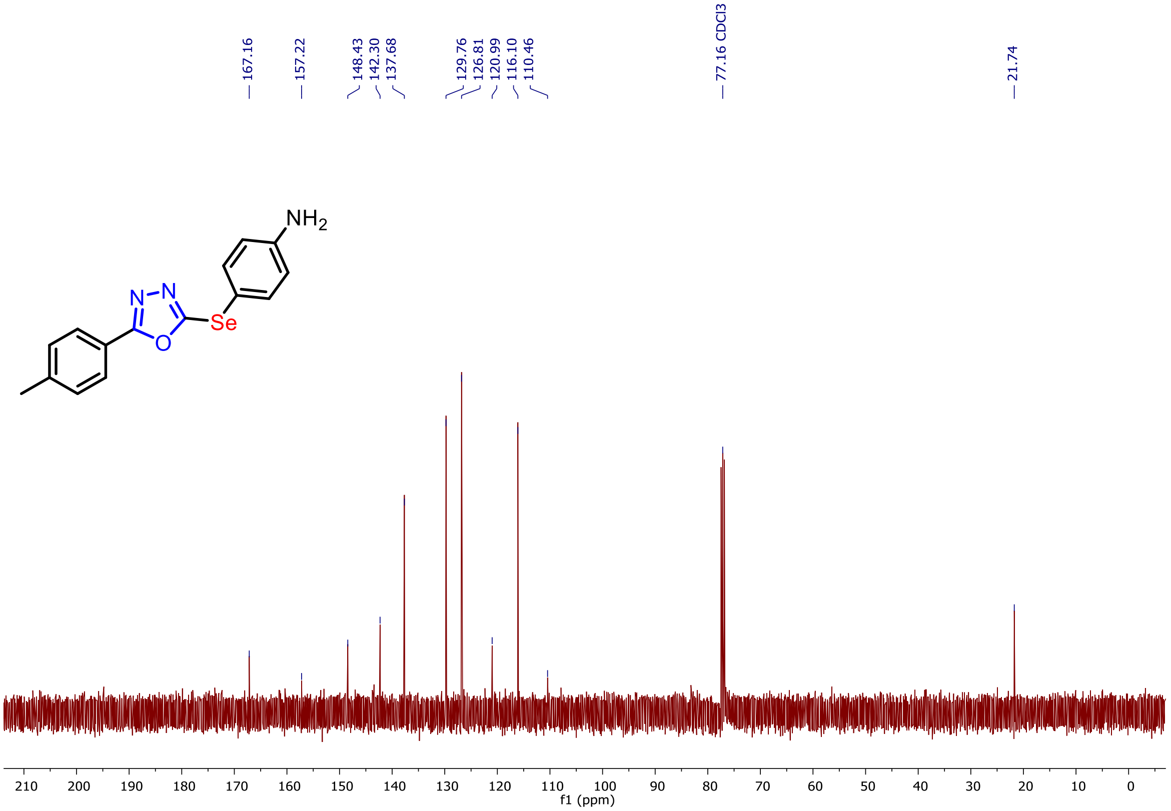

^{13}C NMR (100 MHz, $CDCl_3$) spectrum of 3g.

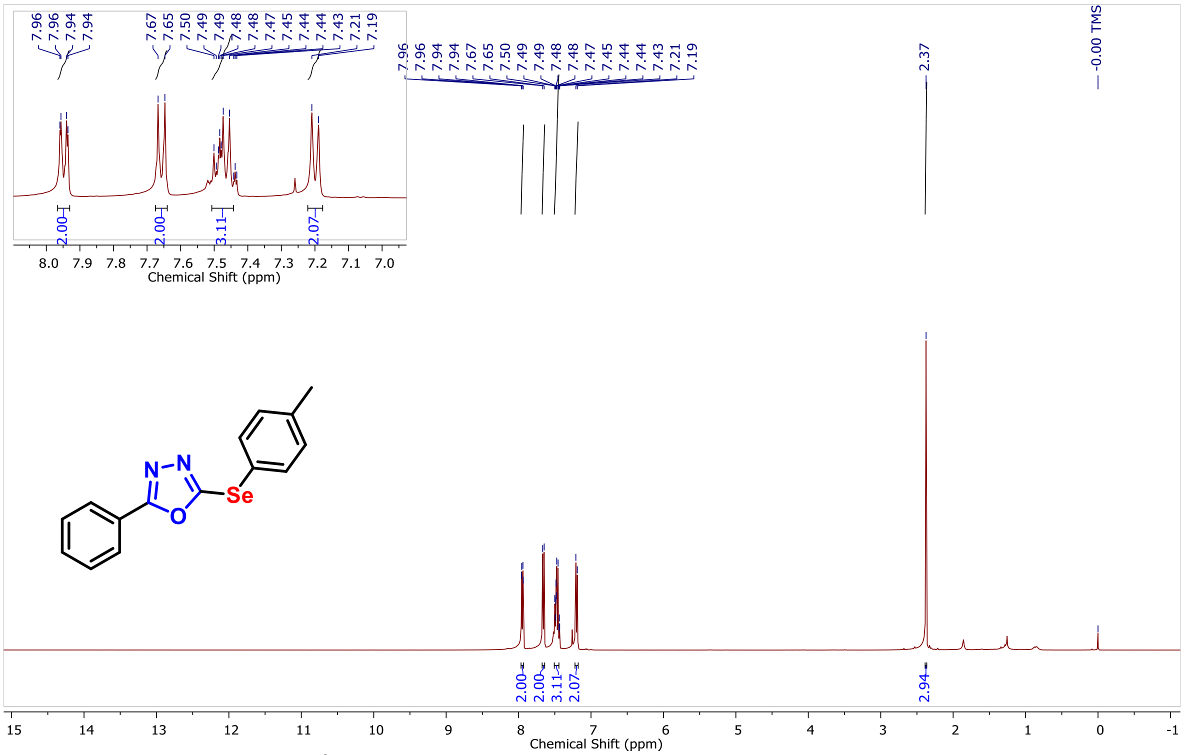

^{1}H NMR (400 MHz, $CDCl_3$) spectrum of 3h.

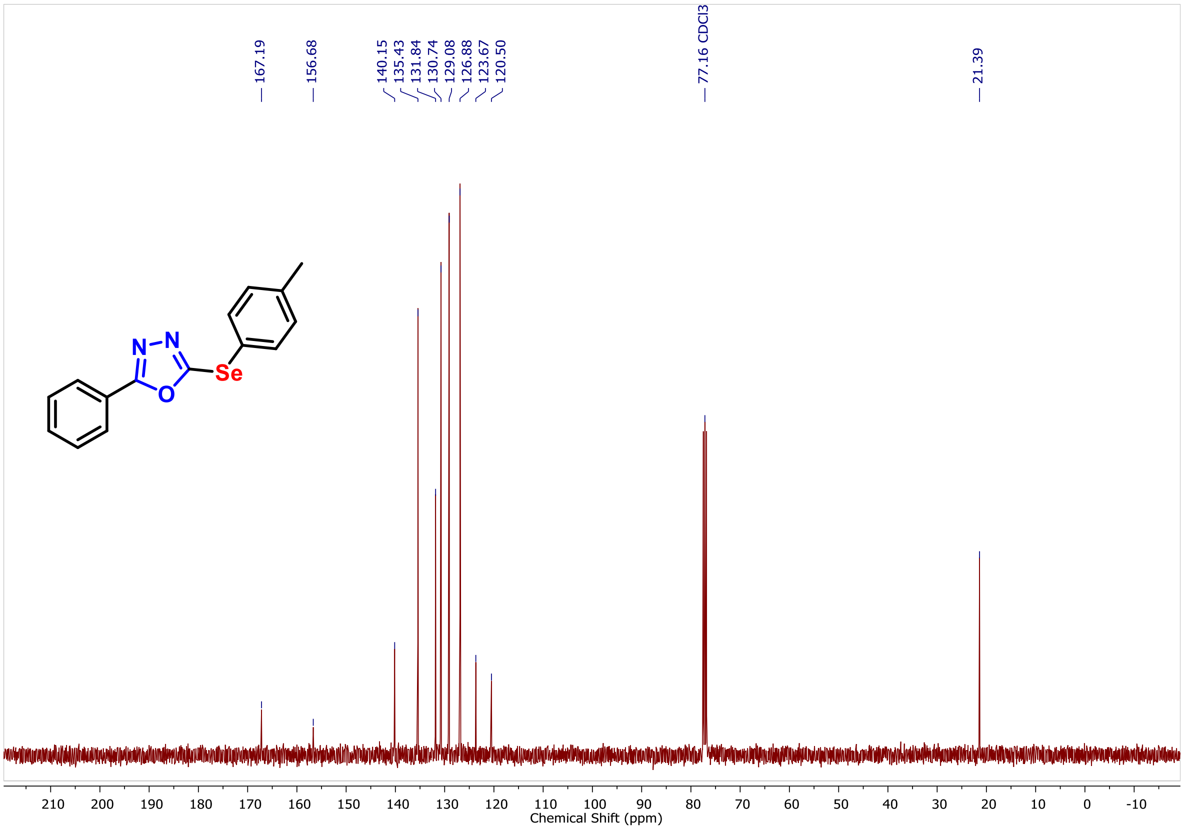

^{13}C NMR (100 MHz, $CDCl_3$) spectrum of 3h.

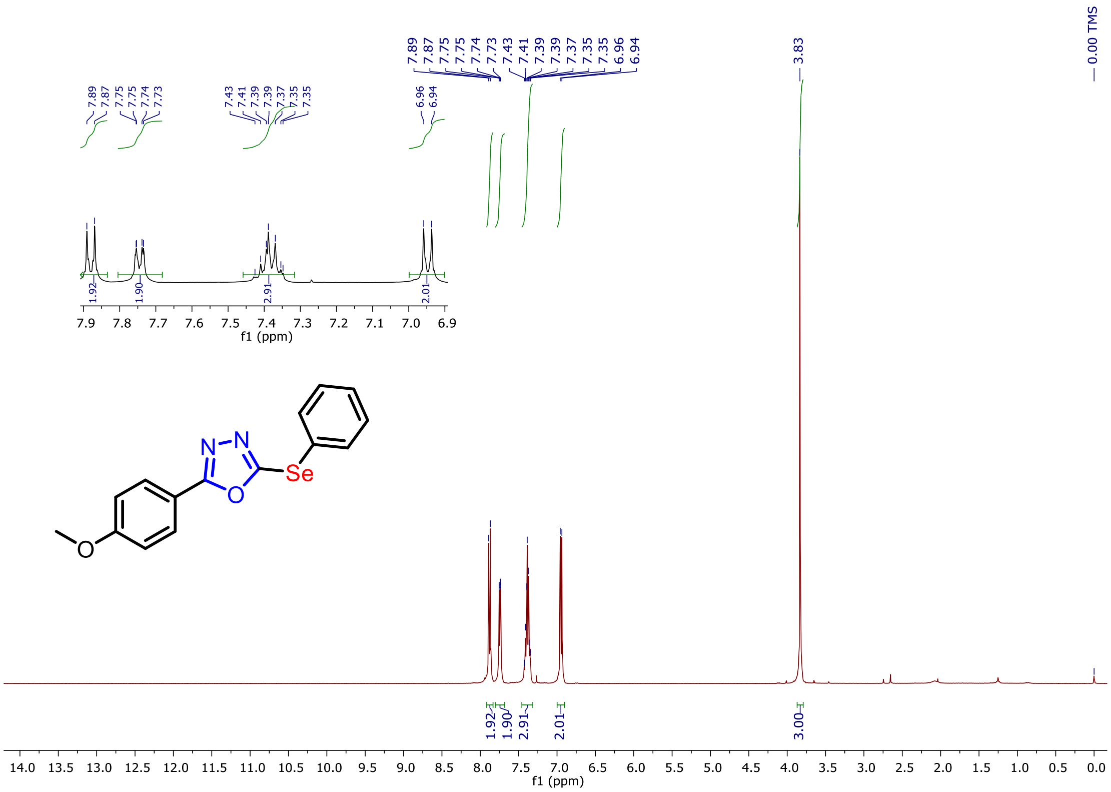

^{1}H NMR (400 MHz, $CDCl_3$) spectrum of 3i.

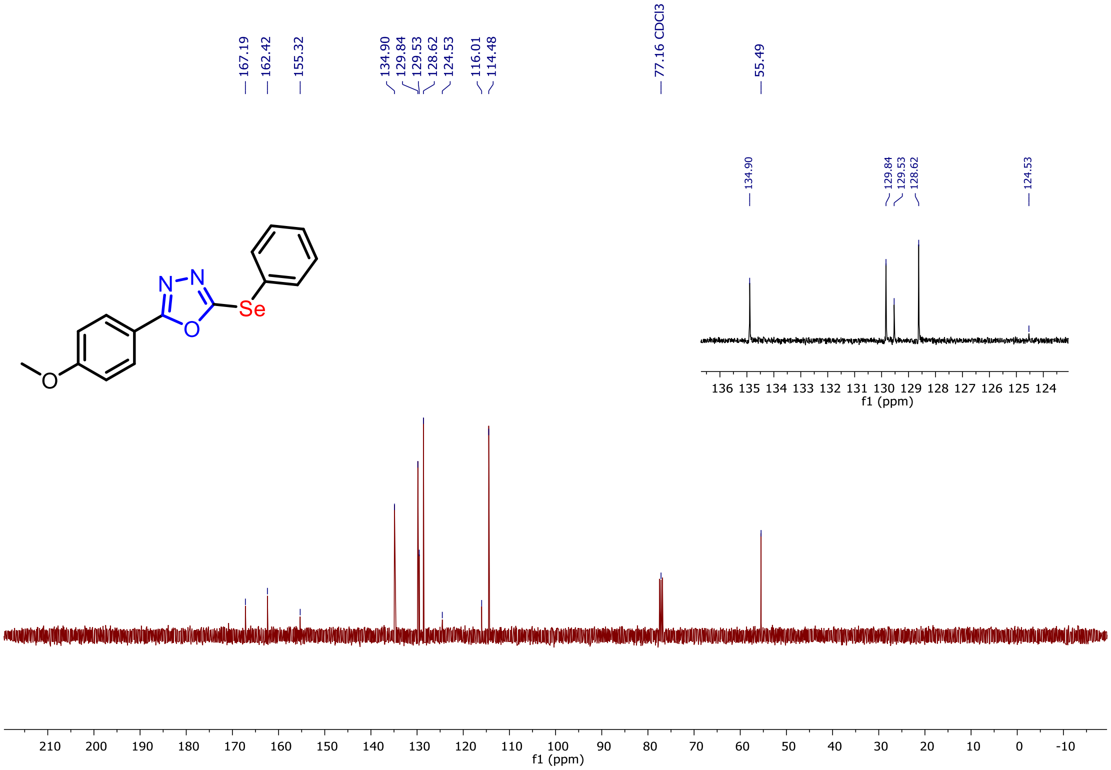

^{13}C NMR (100 MHz, $CDCl_3$) spectrum of 3i.

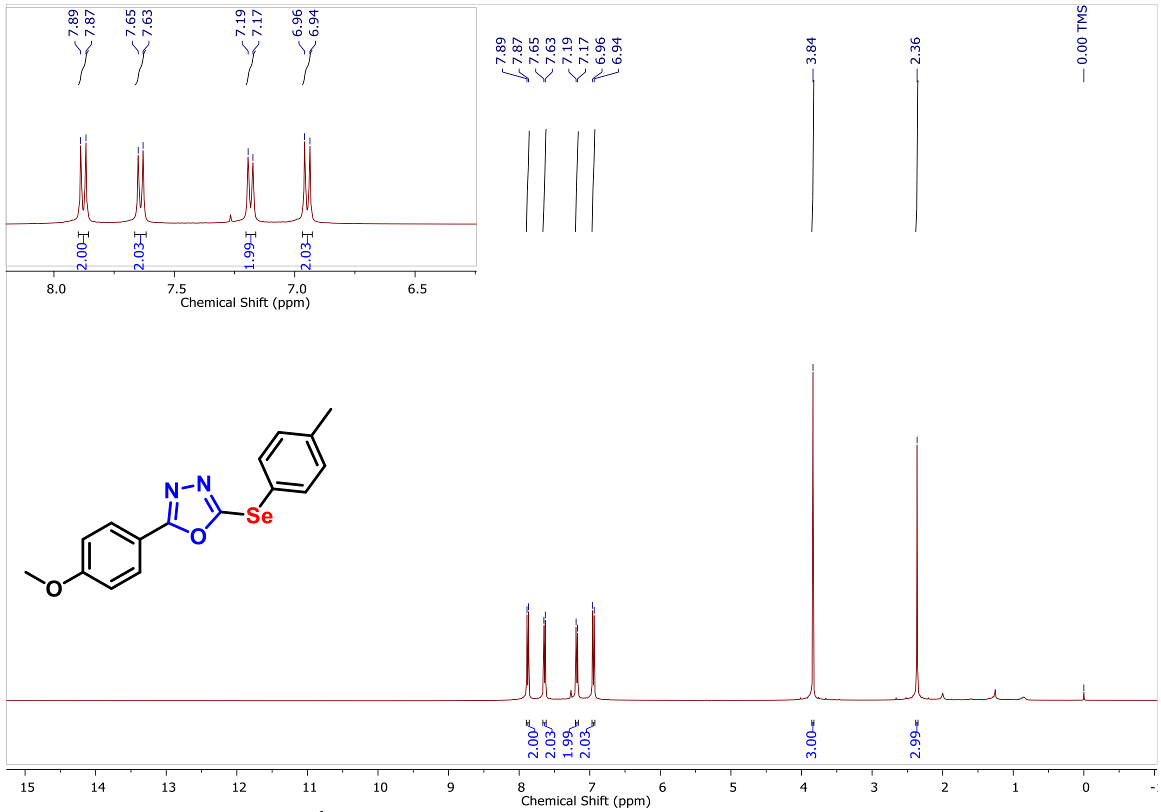

^{1}H NMR (400 MHz, $CDCl_3$) spectrum of 3j.

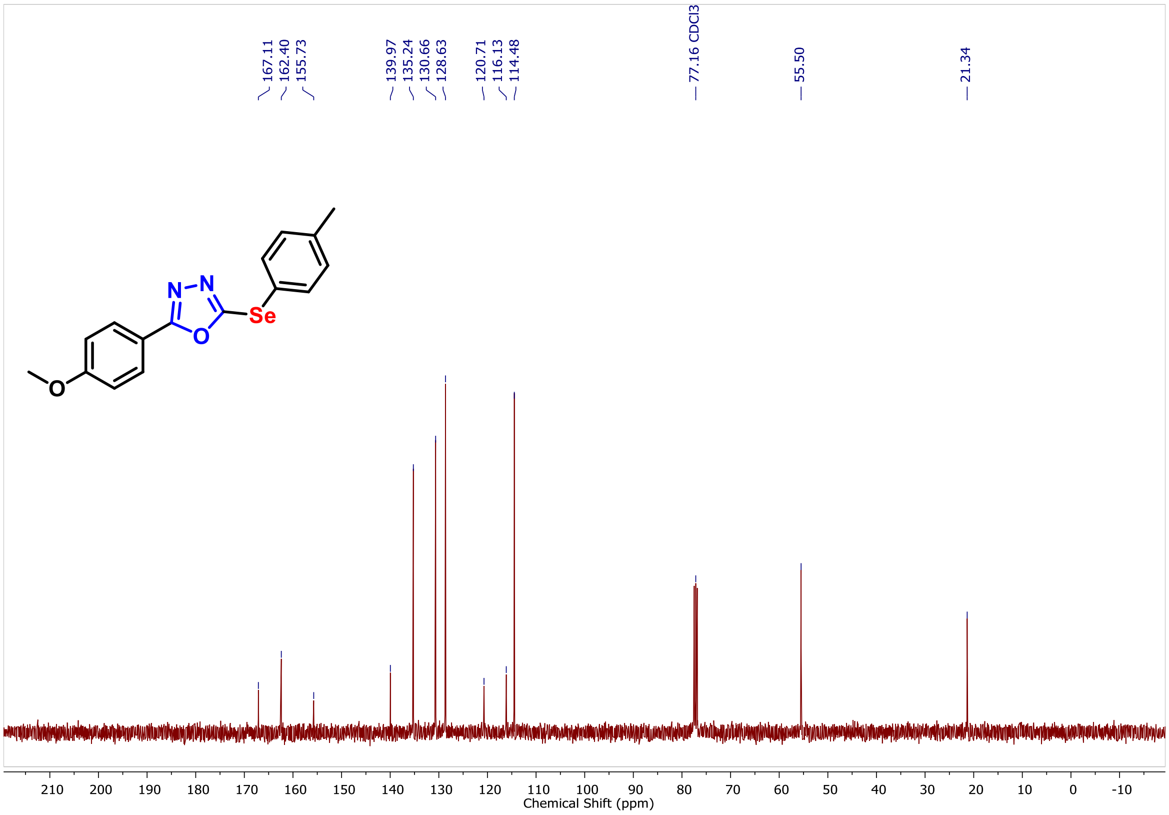

^{13}C NMR (100 MHz, $CDCl_3$) spectrum of 3j

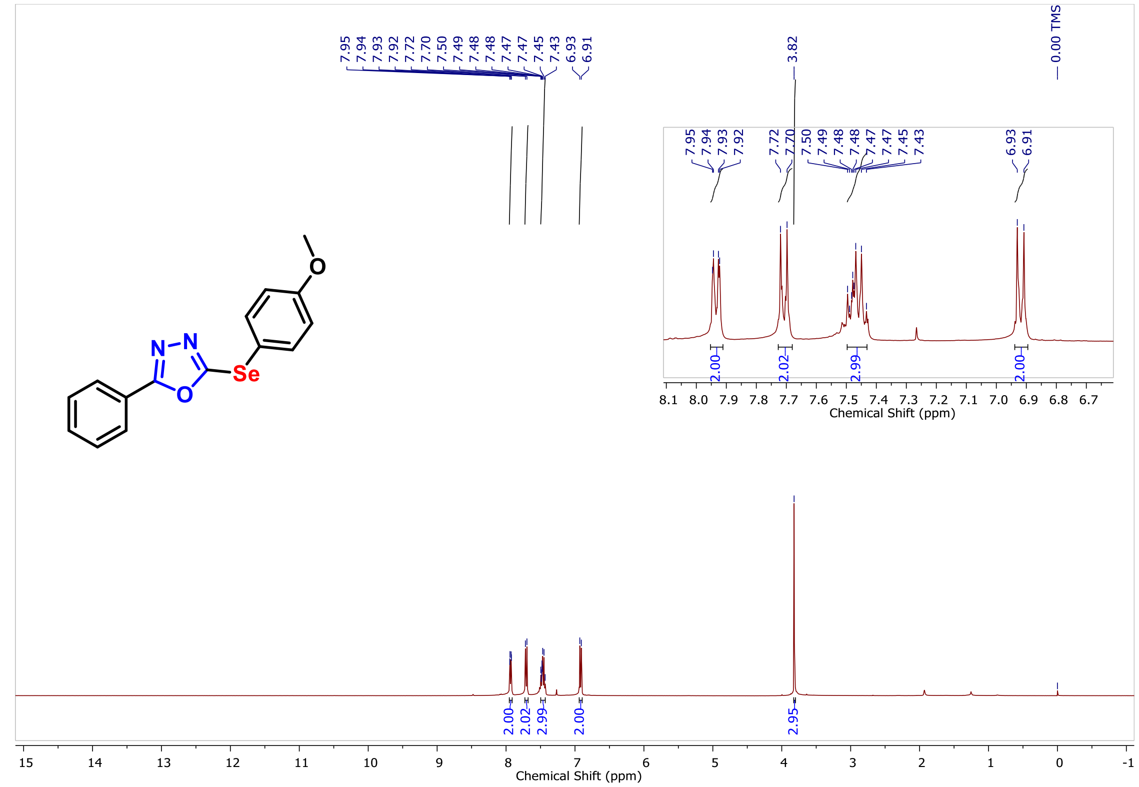

^{1}H NMR (400 MHz, CDCl$_3$) spectrum of 3k.

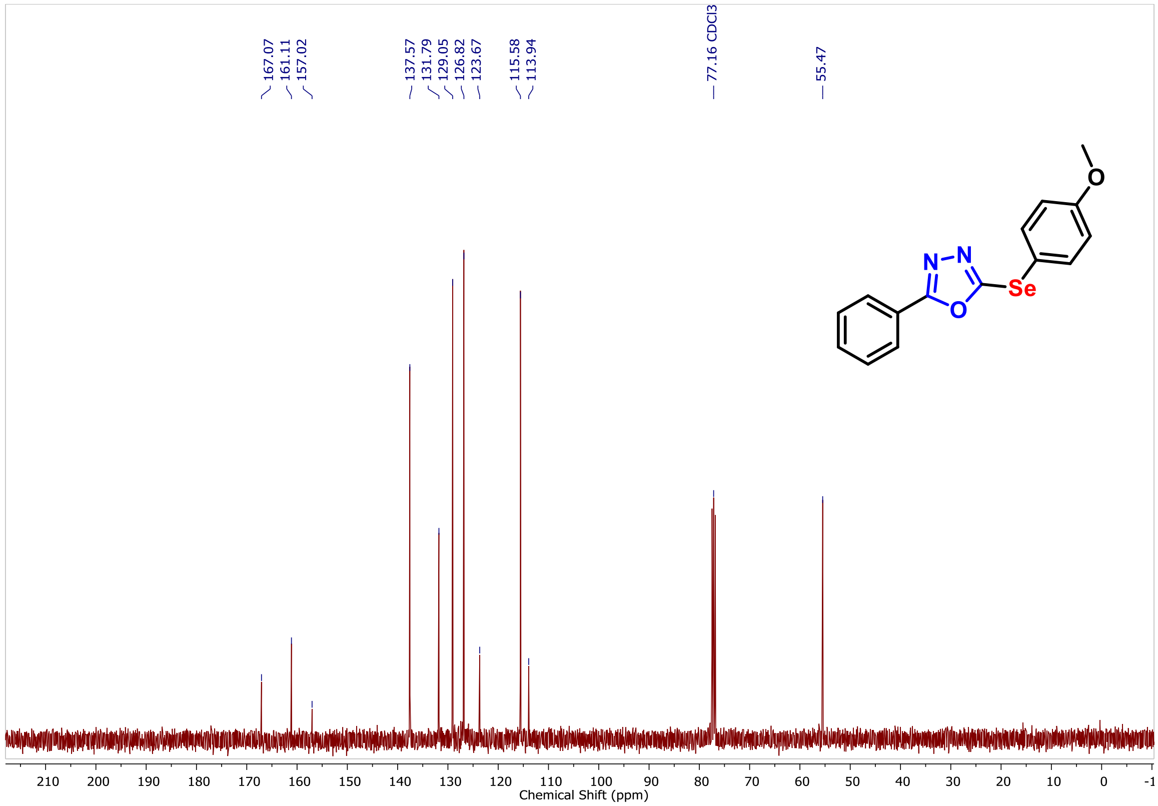

^{13}C NMR (100 MHz, CDCl$_3$) spectrum of 3k.

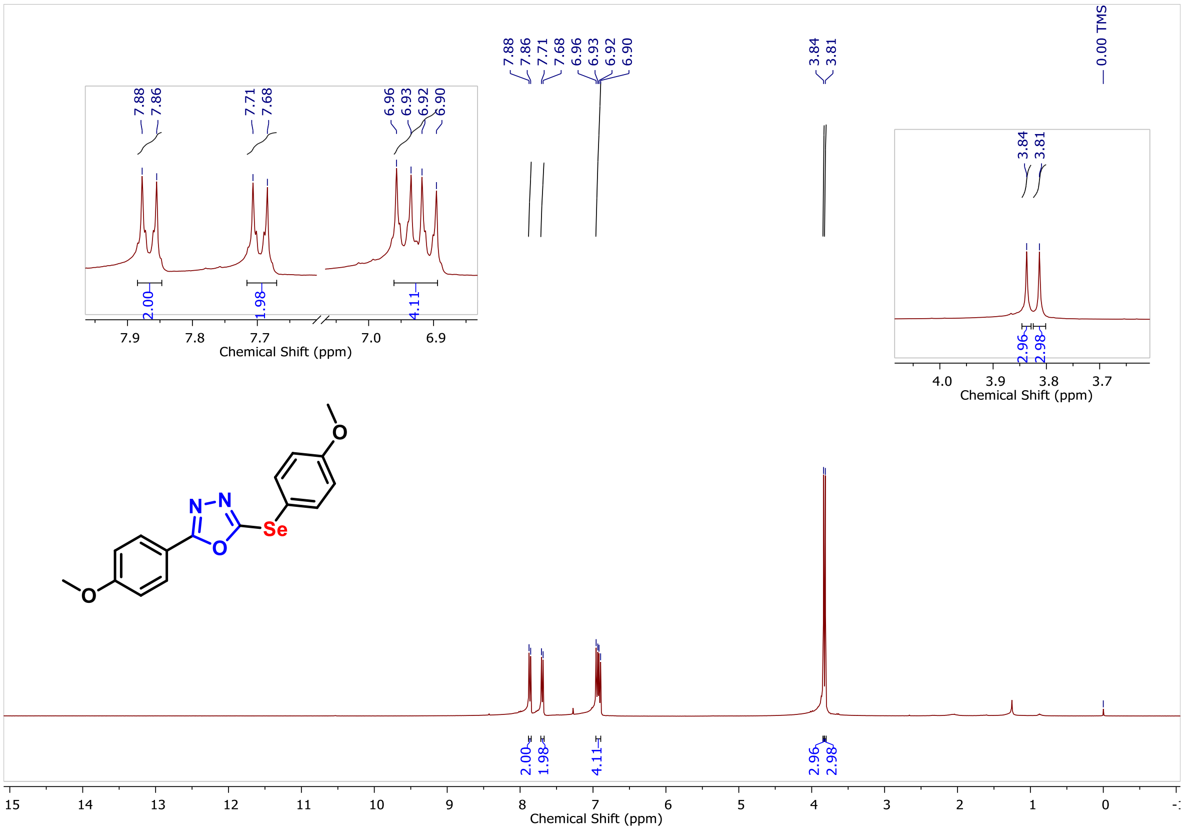

^{1}H NMR (400 MHz, $CDCl_3$) spectrum of 3l.

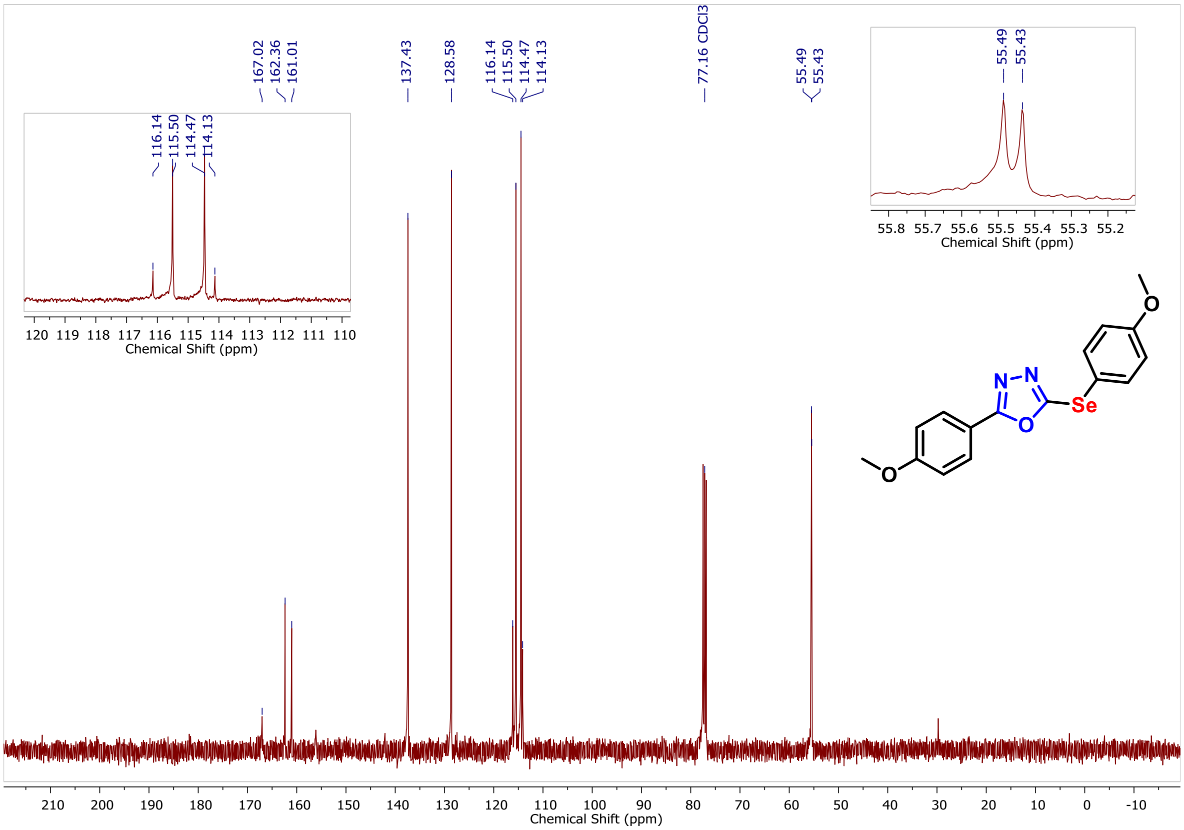

^{13}C NMR (100 MHz, $CDCl_3$) spectrum of 3l.

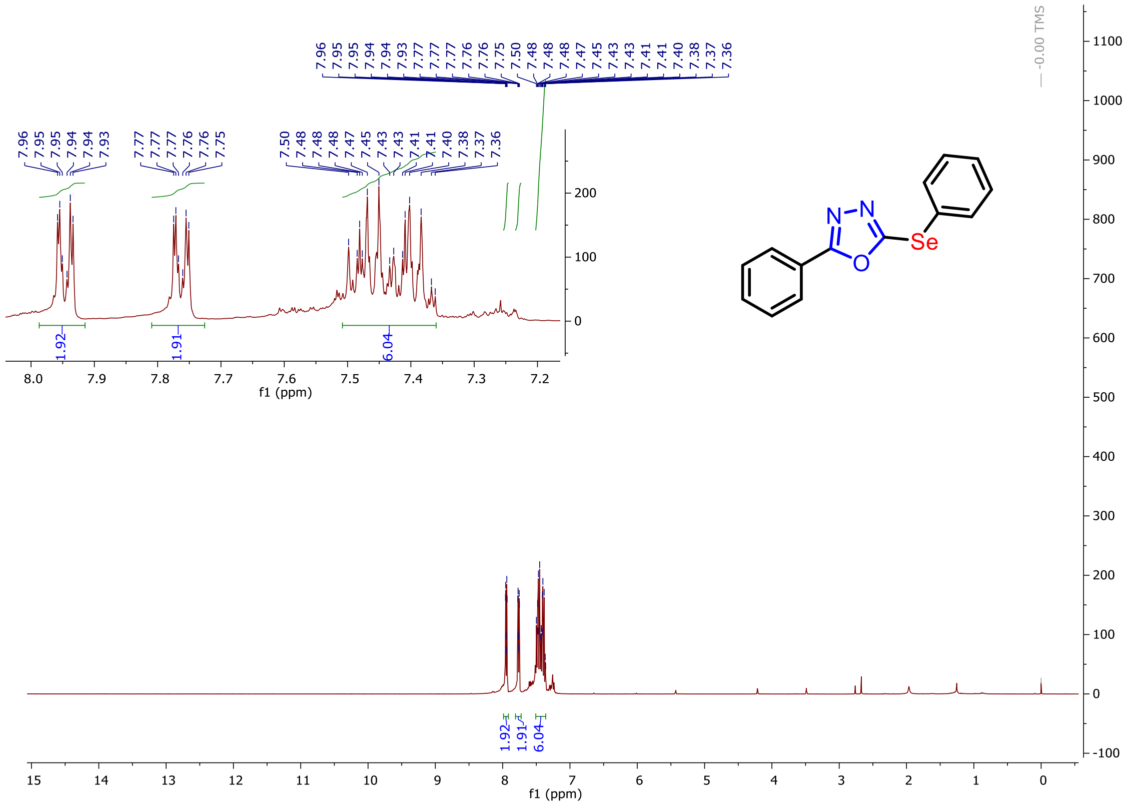

^{1}H NMR (400 MHz, $CDCl_3$) spectrum of 3m.

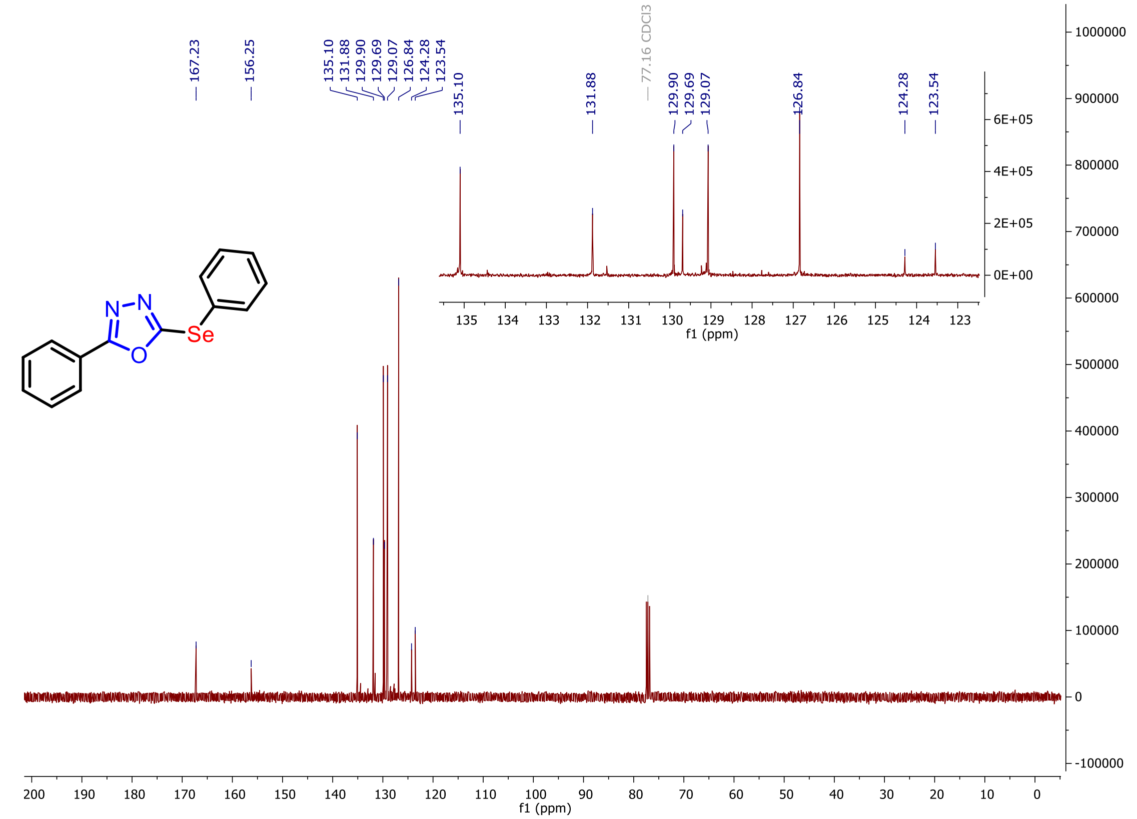

^{13}C NMR (100 MHz, $CDCl_3$) spectrum of 3m.

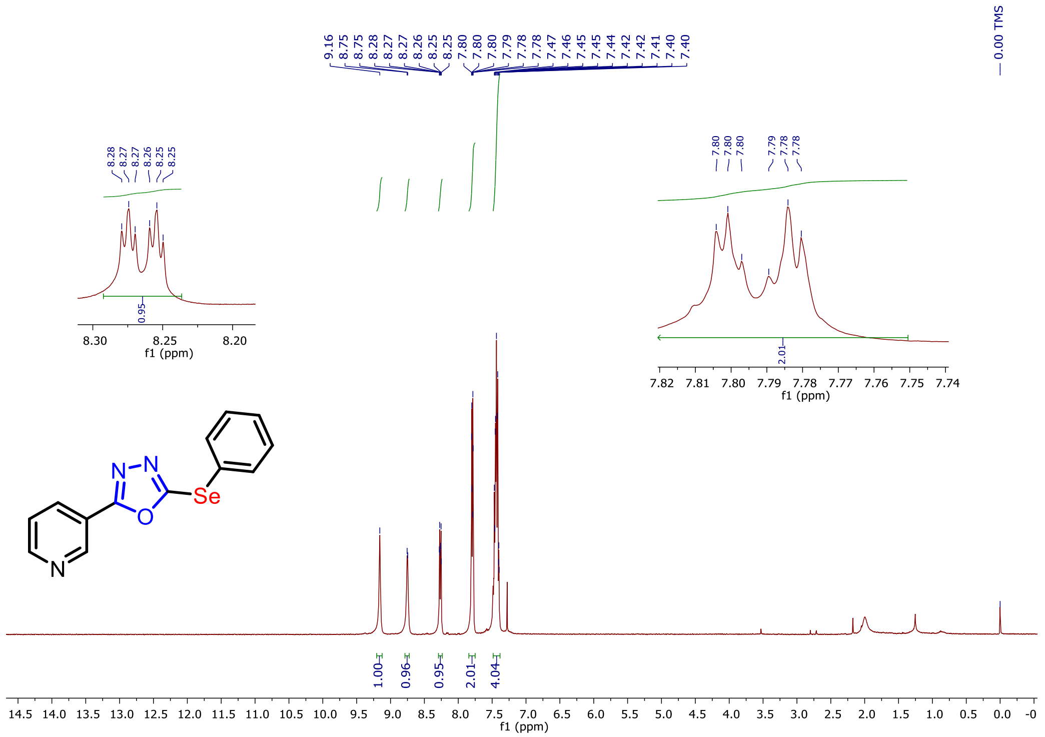

^{1}H NMR (400 MHz, $CDCl_3$) spectrum of 3n.

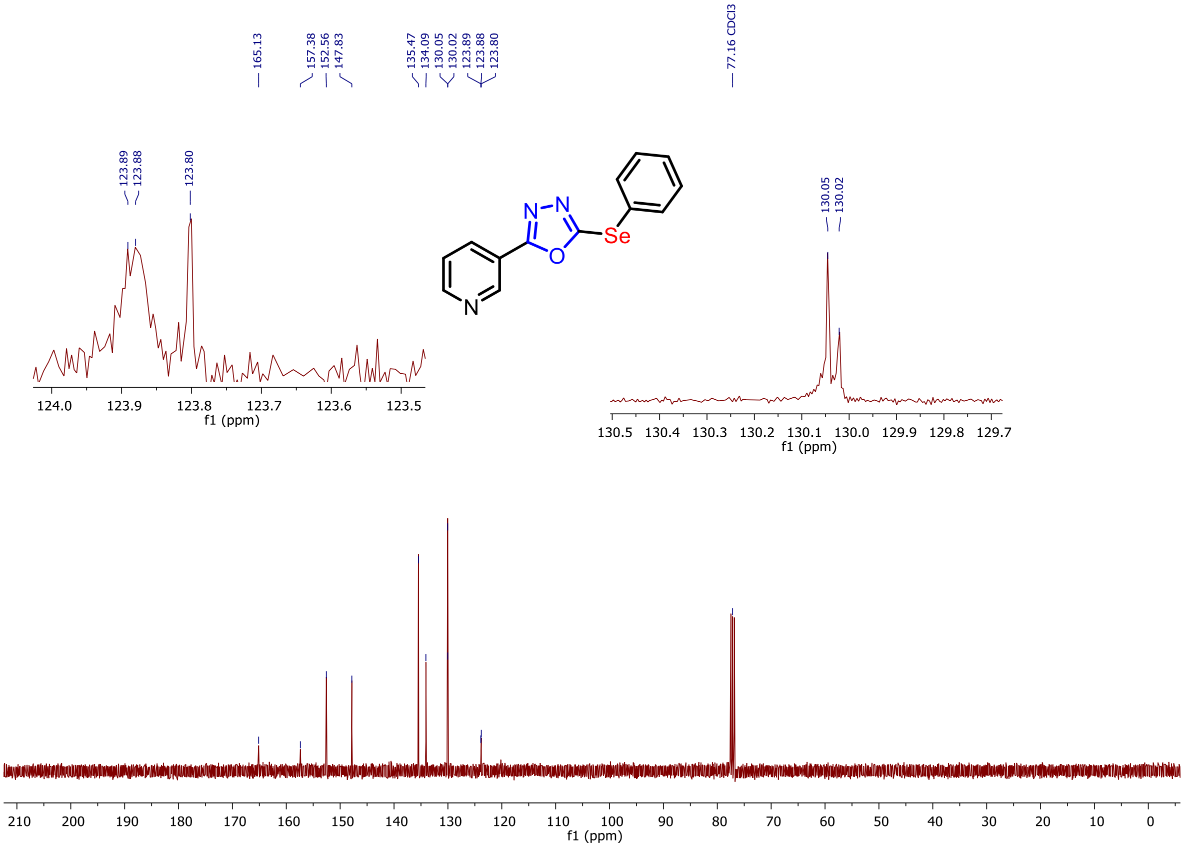

^{13}C NMR (100 MHz, $CDCl_3$) spectrum of 3n.

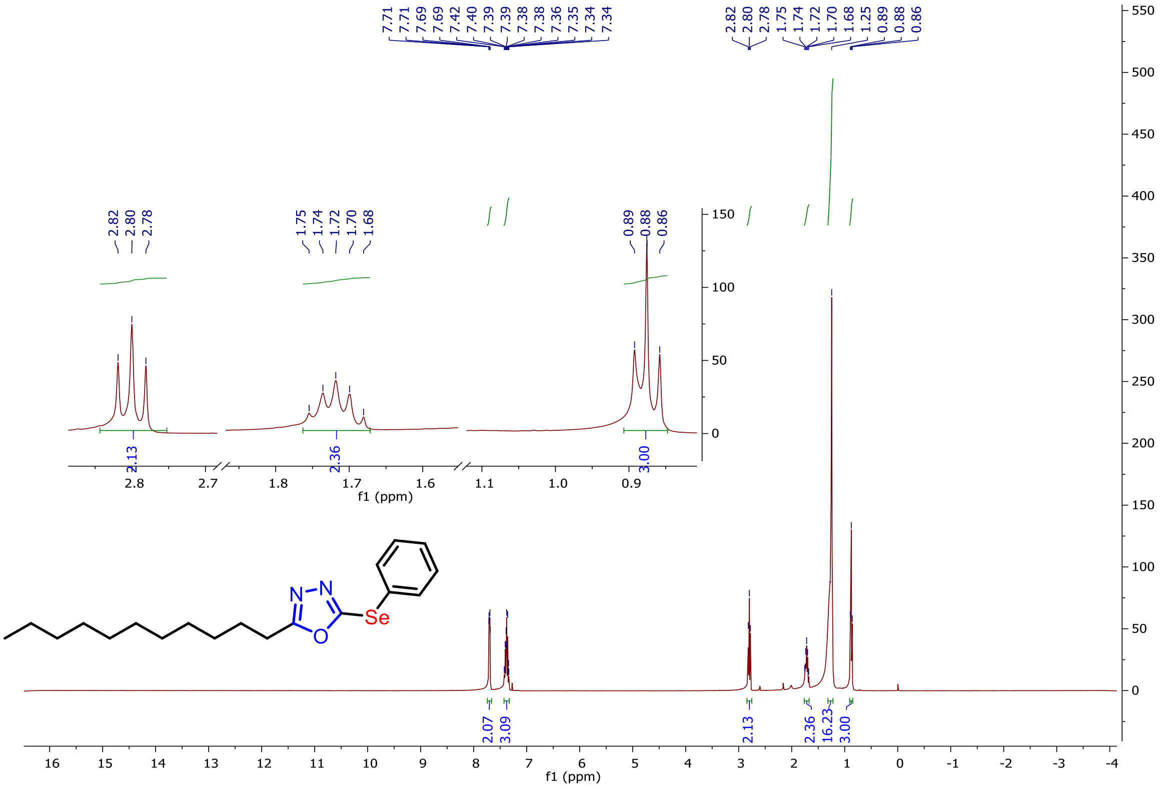

^{1}H NMR (400 MHz, $CDCl_3$) spectrum of 3o.

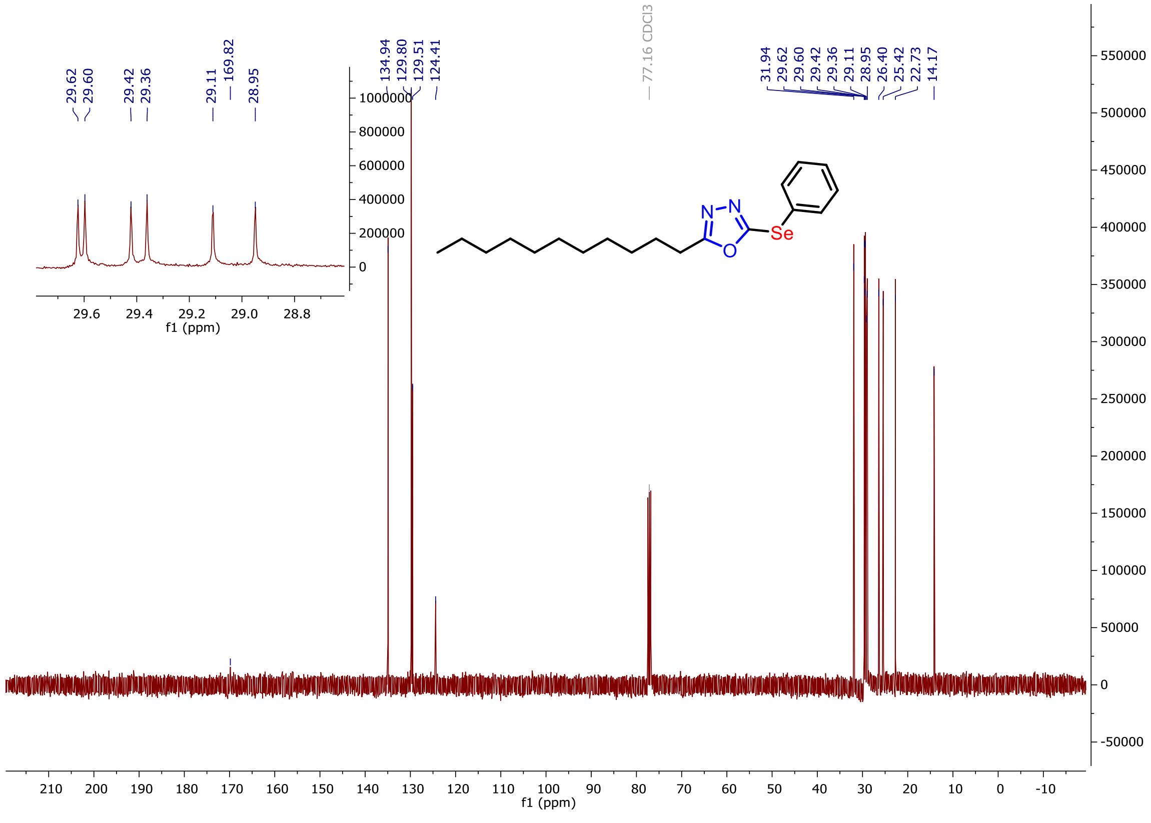

^{13}C NMR (100 MHz, $CDCl_3$) spectrum of 3o.

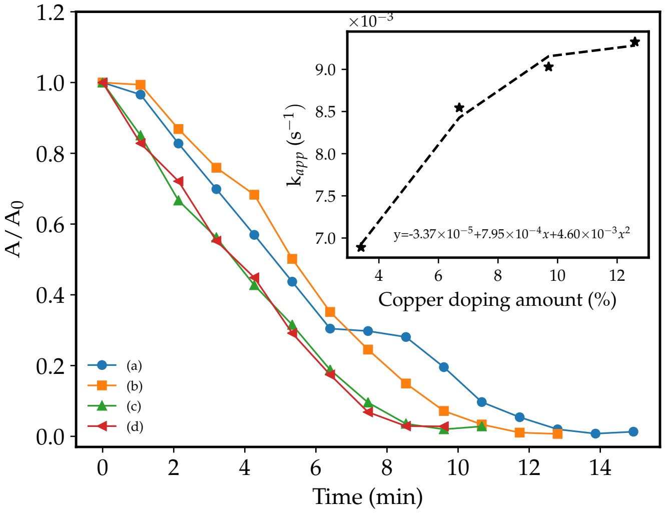

Figure S 3. Typical UV-Vis absorption spectra for 4-nitrophenol reduction by CuOnano@glass catalyst with copper-doped glass matrix (a) 3.4 mol% (0.32 mg of copper), (b) 6.7 mol% (0.65 mg of copper), (c) 9.7 mol% (0.93 mg of copper), and (d) 12.6 mol% (1.22 mg of copper). Graphic in insert: Evolution of kapp as a function of copper amount for four different glass samples.

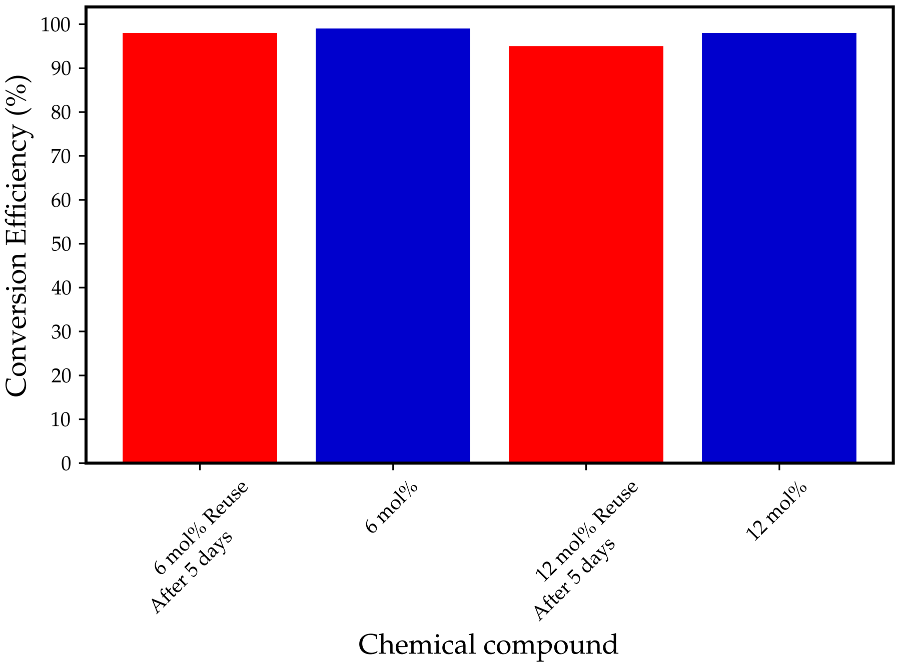

Figure S 4. Effect of catalyst aging, after 5 days, on the catalytic activity of glass-based toward 4-NP reduction for the glass doped with 6 mol% and 12 mol% of copper ions, as a model system.
